# Genomic Ascertainment of *CHEK2*-Related Cancer Predisposition

**DOI:** 10.1001/jamanetworkopen.2025.49730

**Published:** 2025-12-15

**Authors:** Sun Young Kim, Jung Kim, Mark Ramos, Jeremy Haley, Diane Smelser, H. Shanker Rao, Uyenlinh L. Mirshahi, Katherine L. Nathanson, Barry I. Graubard, Hormuzd A. Katki, David Carey, Douglas R. Stewart

**Affiliations:** 1 Clinical Genetics Branch, Division of Cancer Epidemiology and Genetics, National Cancer Institute, NIH, Rockville, Maryland; 2 Louisiana State University Health Sciences Center, New Orleans, Louisiana; 3 now with Pennsylvania State University, College of Health and Human Development, State College; 4 Department of Genomic Health, Geisinger, Danville, Pennsylvania; 5 Division of Translational Medicine and Human Genetics, Department of Medicine, Perelman School of Medicine, University of Pennsylvania, Philadelphia, PA and; 6 Abramson Cancer Center, Perelman School of Medicine, University of Pennsylvania, Philadelphia; 7 Biostatistics Branch, Division of Cancer Epidemiology and Genetics, National Cancer Institute, National Institutes of Health, Rockville, Maryland

## Abstract

**Importance:**

There is clear evidence that deleterious germline variants in *CHEK2* increase risk for breast and prostate cancers; there is limited or conflicting evidence for other cancers.

**Objective:**

To quantify the prevalence of as well as cancer risk and survival associated with *CHEK2* germline pathogenic and likely pathogenic variants using genomic ascertainment.

**Design, Setting, and Participants:**

This case-control study used 2 electronic health record–linked and exome-sequenced biobanks: UK Biobank (n = 469 765) and Geisinger MyCode (adults only; n = 167 050). Variants were classified according to American College of Medical Genetics and Genomics and the Association for Molecular Pathology criteria. Cases were defined as individuals with heterozygous *CHEK2*, harboring pathogenic or likely pathogenic variants; controls as individuals with a benign or likely benign *CHEK2* variation or wildtype *CHEK2*. Cancer registry (MyCode since approximately 1943; UK Biobank since approximately 1970) and demographic data were retrieved; to adjust for relatedness, association analysis was performed with SAIGE-GENE+ with Bonferroni correction.

**Main Outcomes and Measures:**

Prevalence of as well as cancer risk and survival in adults with *CHEK2* germline variants.

**Results:**

Of 469 765 individuals in the UK Biobank, there were 3232 case participants (mean [SD] age, 70.8 [8.0] years; 3139 [97.1%] White; 1744 [54.0%] women); of 167 050 individuals with MyCode, there were 3153 case participants (mean [SD] age, 60.5 [17.8] years; 3123 [98.8%] White; 1935 [61.5%] women). In case participants in both MyCode and UKBB, there was a significant excess risk of all cancers (odds ratio [OR], 1.33 [95% CI, 1.18-1.49]; OR, 1.41 [95% CI, 1.26-1.59], respectively), breast (OR, 1.54 [95% CI, 1.18-2.00]; OR, 1.84 [95% CI, 1.49-2.27], respectively), prostate (OR, 1.62 [95% CI, 1.27-2.07]; OR, 1.78 [95% CI, 1.48-2.16], respectively), kidney (OR, 1.58 [95% CI, 1.03-2.41]; OR, 1.84 [95% CI, 1.22-2.77], respectively), and bladder (OR, 1.50 [95% CI, 1.01-2.23]; OR, 1.64 [95% CI, 1.17-2.31], respectively) cancers as well as lymphoid leukemia (OR, 2.08 [95% CI, 1.17-3.69]; OR, 2.21 [95% CI, 1.19-4.08], respectively). Compared with control participants, time to cancer in case participants was significantly shorter in both cohorts; no significant difference was observed between the age-dependent penetrance of truncating and missense variants for cancer in either cohort. Overall survival was significantly decreased in case participants in UK Biobank; however, the primary consequence was seen after 75 years. There was no statistical difference in survival in MyCode. There were no differences in survival between case participants with cancer and control participants with cancer.

**Conclusions and Relevance:**

In this case-control study of genomic ascertainment of individuals with heterozygous *CHEK2* pathogenic or likely pathogenic variants in 2 population-scale cohorts, there was a significant excess risk of breast, prostate, kidney, bladder, and lymphoid leukemia cancer. The conferred excess mortality and cancer risk was low (ORs <2). This has clinical implications for individuals ascertained this way (vs with a family history of cancer).

## Key Points

**Question:**

What is the cancer risk in adults who harbor a pathogenic or likely pathogenic germline *CHEK2* variant when ascertained genomically?

**Findings:**

In a case-control study of 2 large population- and health system–based cohorts with a total of 636 815 participants, individuals with heterozygous *CHEK2* variants had a significantly increased risk for all cancer; breast, kidney, bladder, and prostate cancer; and lymphoid leukemia, although cancer risk was generally lower compared with phenotypically ascertained cohorts. There were no significant differences in survival between *CHEK2* case participants with cancer vs control participants with cancer.

**Meaning:**

These findings suggest that the method of ascertainment matters when estimating risk and should be considered in clinical decision-making.

## Teaser

This case-control study evaluates the prevalence, cancer risk, and survival among adults with pathogenic or likely pathogenic germline variants in *CHEK2*.

## Introduction

*CHEK2* (OMIM 604373) is a tumor-suppressor gene that is involved in DNA repair in response to cellular DNA damage.^1^ There is clear evidence that individuals with heterozygous *CHEK2* deleterious germline variants are associated with an increased risk for female breast cancer and prostate cancer, and elevated risks for a variety of other cancers (eg, colorectal, kidney, bladder, leukemia/lymphoma, and thyroid) have been observed.^2,3^ In general, germline pathogenic truncating variants (PTV) (eg, c.1100del p.[Thr367fs]) are associated with an increased risk of cancer. In contrast to PTV, pathogenic missense variants (PMV) in *CHEK2* have more variable consequences, mainly dependent on whether a critical protein domain is affected. According to a study by Dorling et al,^4^ approximately 60% of rare PMV in *CHEK2* are associated with a lower risk of developing cancers compared with PTV. This suggests that the impact of PMV on cancer susceptibility is not uniform but rather depends on the specific location and nature of the variants. Most work on quantifying risk from a germline variant in a cancer-predisposition gene has arisen from the well-established phenotype-first approach, in which individuals and families are ascertained from their clinical presentation.

Genomic ascertainment is the inversion of the traditional phenotype-first approach.^5^ With genomic ascertainment, germline variation of interest is identified, and phenotype status is then obtained from medical records to estimate variant prevalence and disease penetrance and to characterize the phenotype. In principle, this should permit a less biased estimate of the phenotypic spectrum, expressivity, and penetrance of a deleterious variant or set of variants. In this case-control study, we used genomic ascertainment to quantify cancer risk for individuals with heterozygous germline pathogenic or likely pathogenic (P/LP) *CHEK2* variants.

## Methods

### Setting and Study Participants

In this case-control study, we analyzed 2 population-based cohorts (UK Biobank [UKBB] and Geisinger MyCode) to estimate the prevalence, age-dependent penetrance, cancer risk, and survival of individuals with heterozygous *CHEK2* P/LP variants (case participants) compared with control participants (with non–P/LP *CHEK2* germline variation). Geisinger is an integrated health system serving patients in northeastern and central Pennsylvania, and patients are eligible to participate in the MyCode Community Health Initiative, a system-wide biorepository of blood and DNA samples for broad research purposes.^6^ The UKBB is a population-scale biobank.^7^

MyCode participants agree that their samples and data can be linked to Geisinger EHRs; additional informed consent for this study beyond the initial written consent was deemed not to be required per Geisinger Institutional Review board. For the UK Biobank, human participant protection and review was through the North West Multi-Centre Research Ethics Committee. This study followed Strengthening the Reporting of Observational Studies in Epidemiology (STROBE) reporting guideline. Additional details are described in eMethods in Supplement 1.

### Sequencing and Relatedness

For UKBB, germline variants were obtained from field 23157, population-level exome OQFE variants, and pVCF format (accessed January 2023). Exome sequencing on UKBB samples has been previously described.^7,8^ The number of unrelated participants was determined by R package ukbtools, using the ukb_gene_samples_to_remove function.

MyCode DNA samples were exome sequenced by the Regeneron Genetics Center as previously described.^9^ In the MyCode cohort, we included individuals older than 18 years (n = 167 050). To remove related individuals while maintaining the largest possible cohort, kinship pairs up to third degree relatives (minimum PI_HAT, 0.1875) were used to create a graph of all relatives.

### Variant Filtering and *CHEK2* Pathogenicity Classification

All variants that pass quality metrics were annotated using snpEFF,^10^ ANNOVAR,^11^ ClinVar^12^ (database retrieved September 23, 2022), and InterVar version 2.1.3.^13^ Variants were classified as P, LP, variant of uncertain significance (VUS), likely benign (LB), and benign (B) using guidelines from the American College of Medical Genetics and Genomics and the Association for Molecular Pathology (ACMG/AMP).^14^ Case participants were defined as individuals who harbored a *CHEK2* P/LP variant, whereas control participants included individuals who harbored canonical or B/LB *CHEK2* variation. In this analysis, the all-variant group refers to individuals with all *CHEK2* P/LP variants, PTV refers to those with predicted *CHEK2* truncating P/LP variants, and PMV refers to those with pathogenic missense *CHEK2* P/LP variants. Additional details are in eMethods in Supplement 1.

### Cancer Phenotype and Vital Status Query

Demographic data (age, sex, body mass index [BMI], alcohol consumption, smoking history, and self-reported race) were obtained for both case and control participants. All racial groups that were enrolled in the 2 biobanks at the time the data were accessed were included. There were no exclusions on the basis of race or ethnicity. Due to small sample size, groups other than White individuals were collapsed. Clinical phenotypes of neoplasms were obtained from cancer registry for MyCode using *International Statistical Classification of Diseases, Tenth Revision, Clinical Modification *(*ICD-10-CM*) codes. The Cancer Registry (fields 40006 and 40013) and Death Registry data for UKBB (field 40001) were queried using *ICD-9* and *ICD-10* codes.

### Statistical Analysis

Demographic data comparisons were completed using Student *t* test for continuous variables and Fisher exact test for binary variables. Power estimates were performed by adapting formulas from Chow et al^15^ to a cohort study setting with the assumption of nonbiased ascertainment. Cancer prevalence was modeled using logistic regression with carrier status for all, PTV, and PMV as the main set of explanatory variables and age, sex, smoking history, alcohol consumption, and BMI as covariates. Kaplan-Meier survival analyses were used to estimate all-cause mortality, penetrance of P/LP *CHEK2* variants for cancer, and overall survival for individuals with cancer in MyCode and UKBB cohorts. Hazard ratios were computed using the Cox proportional-hazards (Cox PH) model, adjusting for age, self-reported race, sex, smoking history, alcohol consumption, and BMI, using the log-rank test for equality to compare differences between the curves for control and case groups. Cox PH also adjusted for relatedness by clustering genetically inferred family units. All the analyses were conducted using R version 4.1.2 (R Project for Statistical Computing). Bonferroni correction was applied to organ-system groupings and not specific cancer types; otherwise, *P* < .05 was the level of statistical significance. Additional details on statistical methods are in eMethods in Supplement 1.

## Results

### Prevalence and Demographic Characteristics of Case Participants With All, PTV, and PMV *CHEK2* Variants in MyCode and UKBB

There were 469 765 individuals in the UKBB cohort and 167 050 individuals in MyCode. eTable 5 in Supplement 2 shows the prevalence of case participants with all variants, PTV, and PMV in both cohorts. eTable 1 in Supplement 2 provides details on the variants. Overall, there were 3232 case participants (mean [SD] age, 70.8 [8.0] years; 1744 [54.0%] women) in UKBB and 3153 case participants (mean [SD] age, 60.5 [17.8] years; 1935 [61.5%] women) in MyCode (eTable 3 in Supplement 2). Both groups were predominantly White, with 3139 White participants (97.1%) and 93 participants (2.9%) belonging to additional racial groups in UKBB and 3123 White participants (98.8%) and 30 participants (1.0%) belonging to additional racial groups in MyCode. The relatedness (up to the third degree) of the MyCode and UKBB cohorts was approximately 30% and approximately 10%, respectively; eTable 5 in Supplement 2 also shows the heterozygote prevalence in the unrelated fraction of the 2 cohorts. In the 2 cohorts, we observed a 3-fold difference in all *CHEK2* P/LP variant frequency, which is driven by differences in missense variation frequency, particularly I157T (eTable 5 in Supplement 2 ). Therefore, we investigated the frequency of 6 common *CHEK2* variants in gnomAD version 4.1, Penn Medicine Biobank (PMBB), and All of Us.^16^ The 2 US-based biobanks (PMBB and All of Us^16^) had similar frequencies for those variants with Geisinger (eTable 2 in Supplement 2). Similarly, the Regeneron Million Exome Variant Browser version 1.1.3^17,18^ showed a greater than 10-fold difference in allele frequency for *CHEK2* I157T in populations in Northern Europe vs the British Isles. eTable 3 in Supplement 2 lists demographic and covariate data for case participants with all variants, PTV, and PMV as well as control participants.

### Risk for Cancer in Both MyCode and UKBB

**Figure 1.**
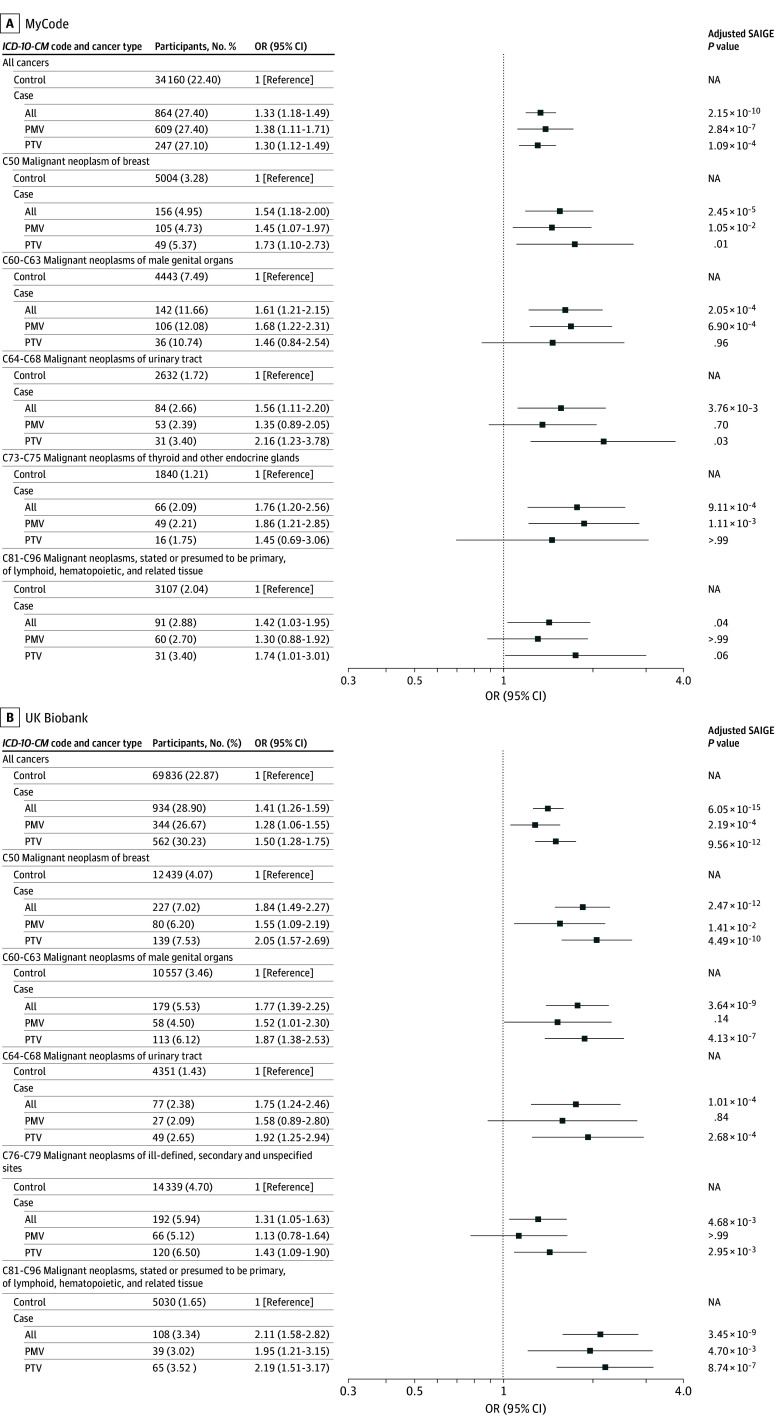
Cancer Risk by Organ System for Case Participants in Both Cohorts Odds ratio (ORs) for case participants with any pathogenic or likely pathogenic variant (all), those with truncating pathogenic or likely pathogenic variants (PTV), and those with pathogenic missense variants (PMV) for organ system groupings of cancer *International Classification of Diseases *codes with a significant excess of risk in MyCode (A) and UK Biobank (B). NA indicates not applicable; SAIGE, Scalable and accurate implementation of generalized mixed model.

Figure 1A displays statistically significant associations for case participants with all variants, PTV, and PMV in *CHEK2 *with organ system groupings of cancer in MyCode. The odds ratios (ORs) and Bonferroni-corrected *P* values for case participants are shown. In case participants in the all-variant group, there was a significant excess risk of all cancers (OR, 1.33 [95% CI, 1.18-1.49]), breast cancer (*ICD-10-CM *code C50: OR, 1.54 [95% CI, 1.18-2.00]), male genital organ cancer (*ICD-10-CM *codes C60-C63), urinary tract cancer (*ICD-10-CM *codes C64-C68), thyroid and other endocrine gland cancers (*ICD-10-CM *codes C73-C75), and lymphoid, hematopoietic, and related tissue cancer (*ICD-10-CM *codes C81-C96). (Of all C50 codes observed in case participants, 155 of 156 [99.4%] and 225 of 227 [99.1%] were in female individuals and 1 [0.6%] and 2 [0.8%] were in males in MyCode and UKBB, respectively.) eFigure 1 in Supplement 1 displays the ORs for the MyCode cohort for all-variant, PTV, and PMV case participant groups for all organ system groupings of cancer *ICD* codes. Figure 1B displays the ORs for the UKBB cohort for all-variant, PTV, and PMV groups of case participants for organ system groupings of cancer *ICD* codes with a significant excess of risk. In the all groups of case participants, there was a significant excess risk of developing all cancers (OR, 1.41 [95% CI, 1.26-1.59]), breast cancer (*ICD-10-CM *code C50; OR, 1.84 [95% CI, 1.49-2.27]), male genital organ cancer (*ICD-10-CM *codes C60-C63), urinary tract cancer (*ICD-10-CM *codes C64-C68), cancer from the secondary and unspecified sites (*ICD-10-CM *codes C76-C79), and lymphoid, hematopoietic, and related tissue cancer (*ICD-10-CM *codes C81-C96). In contrast to MyCode, there was no statistically significant excess risk to develop thyroid and other endocrine gland cancer (*ICD-10-CM *codes C73-C75). eFigure 2 in Supplement 1 displays the ORs for the UKBB cohort for the all, PTV, and PMV case participant groups for all organ system groupings of cancer *ICD* codes.

### Specific Cancers Associated With Case Participants With All Variants, PTV, and PMV in *CHEK2*

**Figure 2.**
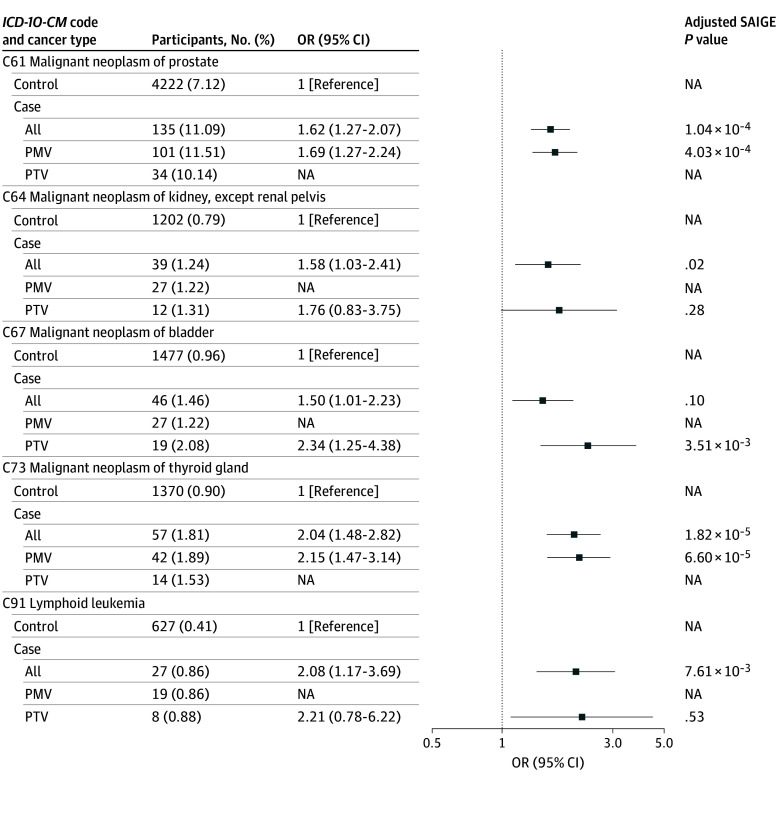
Organ System–Specific Cancer Risks for Case Participants in MyCode Odds ratio (OR) for case participants with any pathogenic or likely pathogenic variant (all), those with truncating pathogenic or likely pathogenic variants (PTV), and those with pathogenic missense variants (PMV) for specific cancers in the organ system groupings of cancer *International Classification of Diseases* codes with a significant excess of risk in MyCode. NA indicates not applicable; SAIGE, Scalable and accurate implementation of generalized mixed model.

Figure 2A shows the specific types of cancer in the MyCode cohort with an excess risk from the significant organ-system analysis shown in Figure 1A. Of note is the significant excess risk for prostate cancer (C61: OR, 1.62 [95% CI, 1.27-2.07]), kidney cancer (C64: OR, 1.58 [95% CI, 1.03-2.41]), bladder cancer (C67: OR, 1.50 [95% CI, 1.01-2.23]), thyroid cancer (C73: OR, 2.04 [95% CI, 1.48-2.82]), and lymphoid leukemia (C91: OR, 2.08 [95% CI, 1.17-3.69]) in the all-variant group of case participants. eFigure 3 in Supplement 1 displays the ORs for the MyCode cohort for all-variant, PTV, and PMV *CHEK2* groups for all specific types of cancer from all organ system groupings of cancer *ICD* codes. eTable 4 in Supplement 2 lists the case counts and percentages for PMV, PTV, and all-variant groups in the case cohort and fold-enrichment (vs controls) for each of the *ICD-10* diagnostic codes in MyCode.

**Figure 3.**
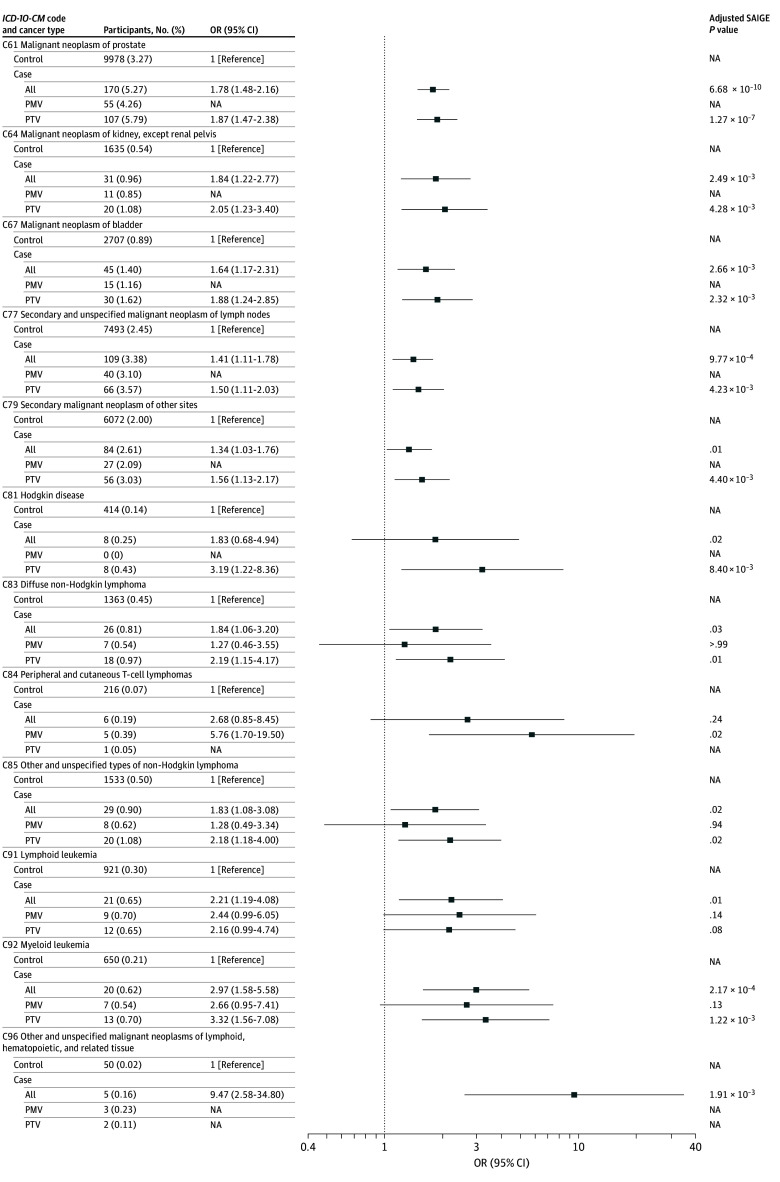
Organ System–Specific Cancer Risks for Case Participants in UK Biobank Odds ratio (OR) for case participants with any pathogenic or likely pathogenic variant (all), those with truncating pathogenic or likely pathogenic variants (PTV), and those with pathogenic missense variants (PMV) for specific cancers in the organ system groupings of cancer *International Classification of Diseases* codes with a significant excess of risk in Biobank. NA indicates not applicable; SAIGE, Scalable and accurate implementation of generalized mixed model.

Figure 3 shows the specific types of cancer in the UKBB cohort with an excess risk from the organ-system analysis shown in Figure 1B. Of note is the significant excess risk for prostate (C61, all-variant group: OR, 1.78 [95% CI, 1.48-2.16]), kidney cancer (C64, all-variant group: OR, 1.84 [95% CI, 1.22-2.77]), and bladder cancer (C67, all-variant group: OR, 1.64 [95% CI, 1.17-2.31]) in all-variant and PTV groups of case participants. There was significant increased risk for diffuse non-Hodgkin lymphoma (C83: OR, 1.84 [95% CI, 1.06-3.20]), other and nonspecified types of non-Hodgkin lymphoma (C85: OR, 1.83 [95% CI, 1.08-3.08]) and lymphoid leukemia (C91: OR, 2.21 [95% CI, 1.19-4.08]) in the all-variant group, whereas peripheral and cutaneous T-cell lymphomas (C84) were exclusively associated with case participants with PMV. eFigure 4 in Supplement 1 displays the ORs for the UKBB cohort for the all-variant, PTV, and PMV *CHEK2* groups for all specific types of cancer from all organ system groupings of cancer *ICD* codes. eTable 4 in Supplement 2 lists the case counts and percentages for PMV, PTV, and all-variant groups for the case cohort and fold-enrichment (vs controls) for each of the *ICD-10* diagnostic codes in UKBB.

### Time to Cancer 

**Figure 4.**
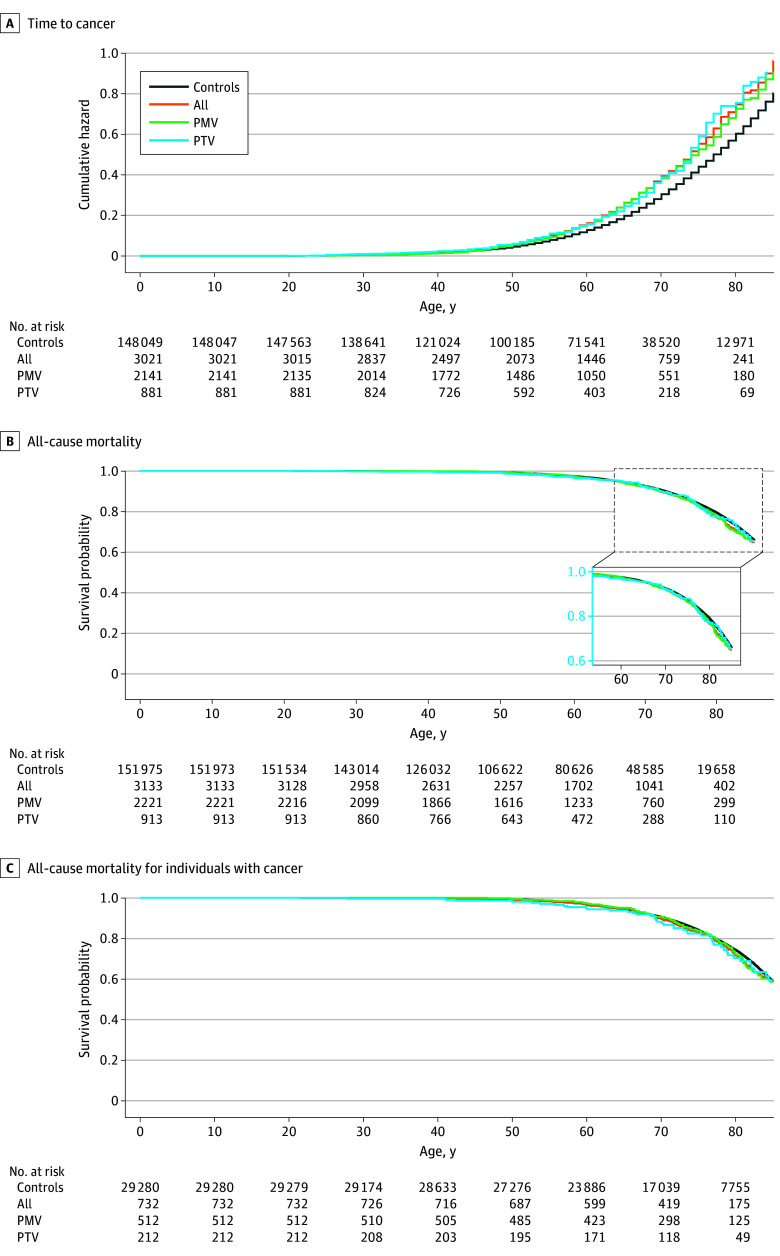
Penetrance of Pathogenic *CHEK2* Variants for Cancer and All-Cause Mortality in MyCode All includes case participants with any pathogenic or likely pathogenic variant; PMV, those with pathogenic missense variants; and PTV, those with pathogenic truncating variant.

**Figure 5.**
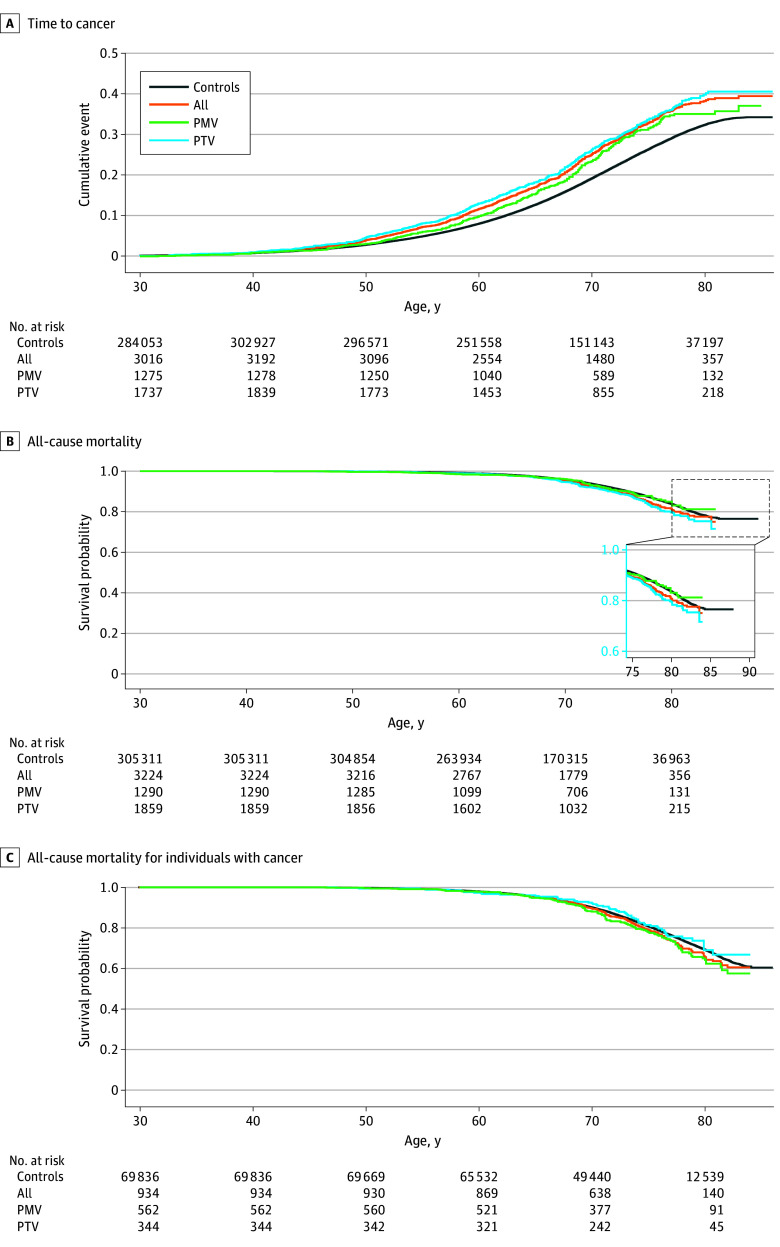
Penetrance of Pathogenic *CHEK2* Variants for Cancer and All-Cause Mortality in UK Biobank All includes case participants with any pathogenic or likely pathogenic variant; PMV, those with pathogenic missense variants; and PTV, those with pathogenic truncating variants.

Compared with controls, time to all cancer in the all group of case participants was significantly different in both MyCode (adjusted HR, 1.26 [95% CI, 1.17-1.36]; *P* < .001) and UKBB (adjusted HR, 1.31 [95% CI, 1.24-1.40]; *P* < .001) (Figure 4A and Figure 5A). Case participants in the PMV group were at higher risk for all cancers tested compared with control participants in both MyCode (adjusted HR, 1.24 [95% CI, 1.13-1.35]; *P* < .001) and UKBB (adjusted HR, 1.17 [95% CI, 1.06-1.30]; *P* = .002). Likewise, case participants in the PTV group were also at higher risk for all cancers tested compared with controls in both MyCode (adjusted HR, 1.30 [95% CI, 1.13-1.50]; *P* < .001) and UKBB (adjusted HR, 1.34 [95% CI, 1.23-1.45]; *P* < .001). There was no significant difference in the penetrance of *CHEK2* PTV vs PMV for cancers in MyCode (univariate HR, 1.06 [95% CI, 0.90-1.25]; *P* = .47) and the UKBB (adjusted HR, 1.15 [95% CI, 1.00-1.33]; *P* = .05).

### All-Cause Mortality

All-cause mortality was significantly increased in the all-variant group of case participants in UKBB, with its consequences observed later in life (age >75 years) (adjusted HR, 1.21 [95% CI, 1.08-1.37]; *P* = .002) but not in MyCode (adjusted HR, 1.09 [95% CI, 0.96-1.24]; *P* = .20) (Figure 4B and Figure 5B). There was no significant difference in all-cause mortality in the PTV and PMV groups in MyCode (adjusted HR, 1.06 [95% CI, 0.80-1.41]; *P* = .67) and UKBB (adjusted HR, 1.24 [95% CI, 0.97-1.60]; *P* = .10).

### All-Cause Mortality Among Individuals With Cancer

There was no statistical difference in all-cause mortality among individuals with cancer between case participants in the all-variant group and control participants in both MyCode (adjusted HR, 1.08 [95% CI, 0.90-1.30]; *P* = .43) and the UKBB (adjusted HR, 1.12 [95% CI, 0.98-1.29]; *P* = .11). There were no significant differences between case participants in the PTV and PMV groups in either the MyCode (adjusted HR, 1.20 [95% CI, 0.81-1.78]; *P* = .35) or UKBB (adjusted HR, 1.33 [95% CI, 0.98-1.80]; *P* = .07) cohorts (Figure 4C and Figure 5C).

## Discussion

Genomic ascertainment quantifies risk based on genotype (not phenotype) and thus may reduce risk inflation arising from cancer ascertainment (case and family recruitment) by personal and/or family medical history. In this investigation, relatedness-adjusted, Bonferroni-corrected genomic ascertainment of 2 population-based, exome-sequenced, electronic health record–linked cohorts was used to quantify cancer risk and survival from P/LP germline variants in *CHEK2*. Both cohorts had high power to detect elevated risk (OR >2) in all but the rarest cancers. The 3-fold difference in *CHEK2* P variant prevalence in the 2 cohorts (driven especially in missense variation) is explained by the known differences in *CHEK2* allele frequencies in US vs British populations.

Clinically, this investigation confirms the significantly increased risk for breast and prostate cancers (as well as all cancers, collectively), although the observed risk tended to be even lower (OR <2) than previous estimates, especially for those in the PTV group (typically OR >2).^2,19^ Interestingly, in neither cohort was a significant excess risk for malignant neoplasms of digestive organs (the majority of which were colorectal cancers) observed for the all-variant, PTV, or PMV groups of case participants (eTable 4 in Supplement 1). Published risk estimates for colorectal cancer from *CHEK2* PTV are more modest (OR of approximately 2) and more conflicting than those for female breast cancer and prostate cancer; higher estimates of risk are driven by studies of multiplex families.^20,21^ Published risk estimates for colorectal cancer from *CHEK2* PMV tend to be even lower (OR <2) or not statistically significant.^2,22^ Given this, a recent ACMG review and clinical practice guideline on management^2^ and current National Comprehensive Cancer Network guidelines (version 3.2024) recommend that *CHEK2* heterozygosity is not clinically actionable for colorectal cancer risk in isolation and to offer surveillance as per family history. In summary, although additional confirmation is needed for breast, prostate, and colorectal cancers, genomic ascertainment showed generally lower (or not significant) risk than previously reported for individuals in the all-variant, PTV, and PMV groups in *CHEK2 *variants.

This work provides substantial evidence from both cohorts of significantly increased risk for kidney cancer, bladder cancer, and chronic lymphocytic leukemia (CLL). In this investigation, Bonferroni correction was applied to organ-system groupings and not specific cancer types. Thus, other cancers may be enriched in individuals with heterozygous *CHEK2* variants; eTable 4 in Supplement 1 lists counts of cancer types in control participants and case participants in the all, PTV, and PMV groups. Several publications have reported increased risk of kidney cancer,^22,23,24,25,26^ whereas other investigations had nonsignificant findings.^27^ As with breast and prostate cancers in this study, the genomic ascertainment used in this study resulted in lower risk estimates (OR <2) for kidney cancer than previous studies and was remarkably consistent across the 2 cohorts. A 2023 ACMG review and clinical guidance for individuals with heterozygous *CHEK2* variants^2^ noted a single publication of nonsignificant *CHEK2*-associated bladder cancer^28^ but deemed this evidence insufficient to make recommendations; more recent publications have found additional evidence of a *CHEK2*-bladder cancer association.^29,30^ Genomic ascertainment in this study revealed similarly increased bladder cancer risk in both cohorts (especially in the PTV groups). Despite the increased relative risk, the absolute risk must also be considered for these rarer cancers. This is especially important in considering actionability of these findings, given the lack of difference in survival between control and case participants in MyCode and differences late in life in UKBB.

In both cohorts there was significantly elevated risk for lymphoid and hematopoietic neoplasms collectively (C81-C96); across all the subtypes of these malignant neoplasms, only CLL had significantly elevated risk (OR >2) in both cohorts. Reports of increased risk of lymphoid and hematologic malignant neoplasms (especially CLL) in individuals with heterozygous *CHEK2* variants date from 2006^21,31,32^ but were conflicting and/or based on highly ascertained families. A 2022 investigation using a phenome-wide association study approach in an earlier version of UKBB reported an excess risk (OR >3) for leukemia and plasma cell neoplasms in individuals with heterozygous *CHEK2* P/LP variants^33^; a 2024 study also using UKBB data found excess risk for Hodgkin lymphoma, diffuse non-Hodgkin lymphoma, and myeloid leukemia in individuals with *CHEK2* PTV.^26^ The role of *CHEK2* P variation may provide clues to the etiology of this leukemia; the clinical actionability of these findings should be considered in the context of minimal differences in survival between control and case participants.

A significant excess of malignant neoplasm of thyroid and other endocrine tumors (C73-C75) was observed in MyCode but not UKBB; this was almost entirely driven by thyroid tumors (C73) and, unlike most other associations, by *CHEK2* PMV. Previous studies have been conflicting or limited by small numbers or single-country ascertainment.^22,27,34^ Genomic ascertainment of *DICER1*-associated thyroid disease (eg, goiter) also found significant differences in individuals with heterozygous *DICER1* variants (vs control participants) in MyCode but not UKBB and may reflect the different medical cultures in the United States and United Kingdom in approaches to medical imaging of the thyroid.^35^ Conversely, there was a significant excess risk of malignant neoplasms of ill-defined, secondary, and unspecified sites (C76-C79) in UKBB but not MyCode.

Numerous other associations have been observed for specific cancers for individuals with heterozygous *CHEK2* variants, including sarcoma, stomach cancer, male breast cancer, melanoma, pancreatic cancer, esophageal cancer, endometrial cancer, and testicular cancer.^2,26^ For more common cancers (eg, endometrial, skin), there was no evidence of association for these in either cohort. For some rarer cancers (male breast, testicular), the 2 cohorts were likely underpowered (eFigure 5 in Supplement 1); for others (sarcoma, stomach), there may be both a power issue and a survival bias in ascertainment given the aggressive nature of these cancers.

### Limitations

There are limitations to these retrospective analyses. Participants in MyCode and UKBB are predominantly of European ancestry. Copy-number variants in *CHEK2* were not evaluated due to limited data availability in UKBB. Enrollment in the 2 cohorts was subject to ascertainment biases, as individuals with conditions leading to death or disabilities would be less likely to participate. The healthy volunteer bias (compared with the UK population) of the UKBB has been documented.^36^ Absolute risk was not quantified.

## Conclusions

In this case-control study, we evaluated cancer risk and survival in individuals with heterozygous *CHEK2* variants using the novel genome-first approach in 2 well-powered cohorts. Pathogenic germline *CHEK2* subgroups of all variants, PTV, and PMV were common in European populations. In addition to breast and prostate risk, we found evidence in both cohorts of associations with kidney and bladder cancers and CLL that may provide clues to etiology. With genomic ascertainment, the conferred excess cancer risk was low (OR <2). This has clinical and counseling implications for individuals ascertained this way (vs with a family history of cancer). In addition, the lack of significant difference between case and control participants in all-cause mortality in individuals with cancer suggests that germline *CHEK2*-associated cancer was not clinically more aggressive than non–*CHEK2*-associated cancer. The degree of risk from PTV and PMV overlap considerably, with risk of PMV generally lower. However, cancer penetrance, all-cause mortality, and all-cause mortality in individuals with cancer was not significantly different between PMV and PTV, suggesting that clinical differences between these variant types are less relevant.

## Notes

Supplement 1.
eMethods.
**eFigure 1.** Odds Ratio for All, PTV, and PMV Groups of Case Participants for Organ System Groupings of Cancer *ICD* Codes in MyCode **eFigure 2.** Odds Ratio for All, PTV, and PMV Groups of Case Participants for Organ System Groupings of Cancer ICD Codes in UK Biobank **eFigure 3.** Odds Ratio for All, PTV, and PMV Groups of Case Participants for All Specific Cancers in the Organ System Groupings of Cancer ICD Codes in MyCode **eFigure 4.** Odds Ratio for All, PTV, and PMV Groups of Case Participants for All Specific Cancers in the Organ System Groupings of Cancer ICD Codes in UK Biobank **eFigure 5.** Power as a Function of Risk (Odds Ratio) in MyCode and UK Biobank for a Range of Cancer Rates

Supplement 2. **eTable 1.** List of all variants found in the study **eTable 2:** Frequency of six common *CHEK2* variants in different biobanks **eTable 3.** Demographics of *CHEK2* heterozygotes vs controls **eTable 4.** Case counts and percentages for PMV, PTV and All cohorts and fold-enrichment (vs controls) for each of the ICD10 diagnostic codes in MyCode and UK Biobank **eTable 5.** Prevalence of All Variants, PTVs, and PMVs in *CHEK2* Among Adults in UK Biobank and Geisinger MyCode

Supplement 3.
Nonauthor Collaborators

Supplement 4.
Data Sharing Statement
