# Supplementary material for: Genomic Ascertainment of CHEK2-Related Cancer Predisposition
Source: JAMA Netw Open. 2025 Dec 15;8(12):e2549730. doi: 10.1001/jamanetworkopen.2025.49730 (PMC12706675; doi:10.1001/jamanetworkopen.2025.49730)
Supplement: Supplement 1. — eMethods. eFigure 1. Odds Ratio for All, PTV, and PMV Groups of Case Participants for Organ System Groupings of Cancer ICD Codes in MyCode eFigure 2. Odds Ratio for All, PTV, and PMV Groups of Case Participants for Organ System Groupings of Cancer ICD Codes in UK Biobank eFigure 3. Odds Ratio for All, PTV, and PMV Groups of Case Participants for All Specific Cancers in the Organ System Groupings of Cancer ICD Codes in MyCode eFigure 4. Odds Ratio for All, PTV, and PMV Groups of Case Participants for All Specific Cancers in the Organ System Groupings of Cancer ICD Codes in UK Biobank eFigure 5. Power as a Function of Risk (Odds Ratio) in MyCode and UK Biobank for a Range of Cancer Rates [file jamanetwopen-e2549730-s001.pdf]

Supplemental Online Content

Kim SY, Kim J, Ramos M, et al; Geisinger-Regeneron DiscovEHR Collaboration. Genomic ascertainment of *CHEK2*-related cancer predisposition. *JAMA Netw Open.* 2025;8(12):e2549730. doi:10.1001/jamanetworkopen.2025.49730

eMethods.

eFigure 1. Odds Ratio for All, PTV, and PMV Groups of Case Participants for Organ System Groupings of Cancer *ICD* Codes in MyCode

eFigure 2. Odds Ratio for All, PTV, and PMV Groups of Case Participants for Organ System Groupings of Cancer ICD Codes in UK Biobank

eFigure 3. Odds Ratio for All, PTV, and PMV Groups of Case Participants for All Specific Cancers in the Organ System Groupings of Cancer ICD Codes in MyCode

eFigure 4. Odds Ratio for All, PTV, and PMV Groups of Case Participants for All Specific Cancers in the Organ System Groupings of Cancer ICD Codes in UK Biobank

eFigure 5. Power as a Function of Risk (Odds Ratio) in MyCode and UK Biobank for a Range of Cancer Rates

This supplemental material has been provided by the authors to give readers additional information about their work.

© 2025 Kim SY et al. *JAMA Network Open*.

eMethods

Setting and study participants

MyCode participants agree that their samples and data can be linked to Geisinger electronic health records (EHRs); additional informed consent for this study beyond the initial written consent was deemed not to be required, as per the Geisinger Institutional Review board. EHR data was available from January 1, 1996 until October 31, 2022; Geisinger Cancer Registry data was available since 1943.

For the UK Biobank, human subjects' protection and review was through the North West Multi-centre Research Ethics Committee. EHR data was available from 1980 through 1996 (ICD9) and 1992 through 2023 (ICD10). Cancer registry data was available from 1967–2024.

Variant filtering and classification

Variants were filtered on the following quality metrics: Allelic Balance of Heterozygotes (ABHet) between 0.2 and 0.8, Genotype Quality >30, total read depth>5.

Final variant annotation was based on a hierarchical classification of ClinVar followed by InterVar[14]. There were eight individuals and six individuals who harbored biallelic *CHEK2* variants in UKBB and MyCode, respectively; they were included in the All group, but were excluded from analyses of PTV and PMV. There was no individual who carried more than two P/LP variants in either UKBB or MyCode.

Cancer-risk and KM analysis

© 2025 Kim SY et al. *JAMA Network Open*.

For initial cancer prevalence, we aggregated cancer ICD10 codes based on ICD10 section. Any significant cancer ICD10 section were extracted and each category was tested. For sex-specific cancers (C51-C58 for female; C60-C63 for male) prevalence was analyzed only with female or male controls. Multiplicity issues were addressed using Bonferroni adjustment at family-wise error rate of $\alpha=0.05$. To further help guard against inaccurate p-values and confidence interval coverage for associations arising from very low prevalence of rare cancers, cancers where there were less than five cases among heterozygotes were excluded from any analyses.

In the MyCode cohort, only events occurring 3 months or after in Geisinger facilities are included in survival analyses. Data was truncated to individuals with current age ≤ 85.

Relatedness correction

Custom functions employing the network library in python were used. For each connected component (i.e., family), the node (i.e., patient) with the greatest number of edges (i.e., relatives) was removed. This was repeated until no edges remain in the connected component.

To correct for relatedness, we used SAIGE-GENE+ version 1.1.6.2. Covariates include using PC1-4, current age, sex, smoking, alcohol use and BMI.[15]

Power estimate

eFigure 5 shows power as a function of presumed true odds ratio for a range of cancer rates in the UK Biobank and MyCode cohorts using cohort-specific All, PTV and PMV *CHEK2* heterozygote prevalence from **Table 1**.

For All, PTV and PMV *CHEK2* heterozygotes, there is 100% power in both UK Biobank and MyCode to detect common cancers (≥5% cancer rate, which include many sex-specific cancers

© 2025 Kim SY et al. *JAMA Network Open*.

such as female breast and prostate) with an odds ratio of >2. For All, PTV and PMV *CHEK2* heterozygotes, there is ≥80% power to detect rare cancers (≥1% cancer rate) with an odds ratio of >2. For All *CHEK2* heterozygotes, there is >80% power to detect very rare cancers (≥0.1%) with an odds ratio of >2.7 in both MyCode and UKBB; there is less power in the PTV- and PMV-specific cohorts.

© 2025 Kim SY et al. *JAMA Network Open*.

eFigure 1. Odds Ratio for All, PTV, and PMV Groups of Case Participants for Organ System Groupings of Cancer *ICD* Codes in MyCode

Controls(%)	Heterozygotes(%)	OR [95% CI]	Adjusted SAIGE p–value	
34,160(22.40)	864(27.40)	1.33[1.18–1.49]	2.15E–10	
34,160(22.40)	609(27.40)	1.38[1.11–1.71]	2.84E–07	All Cancers
34,160(22.40)	247(27.1)	1.30[1.12–1.49]	1.09E–04	
895(0.59)	21(0.67)	1.1[0.56–2.13]	1	
895(0.59)	12(0.54)	0.87[0.37–2.03]	1	C00–C14 Malignant neoplasms of lip, oral cavity and pharynx
895(0.59)	9(0.99)	1.71[0.63–4.65]	1	
4,096(2.68)	109(3.46)	1.27[0.94–1.7]	0.33	
4,096(2.68)	78(3.51)	1.26[0.89–1.78]	0.14	C15–C26 Malignant neoplasms of digestive organs
4,096(2.68)	30(3.29)	1.26[0.72–2.21]	1	
2,791(1.83)	54(1.71)	0.9[0.6–1.37]	1	
2,791(1.83)	38(1.71)	0.88[0.53–1.45]	1	C30–C39 Malignant neoplasms of respiratory and intrathoracic organs
2,791(1.83)	16(1.75)	0.97[0.45–2.1]	1	
256(0.17)	7(0.22)	1.31[0.42–4.09]	1	
256(0.17)	6(0.27)	1.57[0.7–3.54]	1	C40–C41 Malignant neoplasms of bone and articular cartilage
256(0.17)	1(0.11)	NA	NA	
12,443(8.15)	252(7.99)	0.95[0.77–1.17]	1	
12,443(8.15)	174(7.83)	0.9[0.71–1.15]	1	C43–C44 Melanoma and other malignant neoplasms of skin
12,443(8.15)	76(8.32)	1.05[0.72–1.53]	1	
624(0.41)	15(0.47)	1.15[0.53–2.49]	1	
624(0.41)	13(0.59)	1.39[0.8–2.42]	1	C45–C49 Malignant neoplasms of mesothelial and soft tissue
624(0.41)	1(0.11)	NA	NA	
5,004(3.28)	156(4.95)	1.54[1.18–2.00]	2.45E–05	
5,004(3.28)	105(4.73)	1.45[1.07–1.97]	1.05E–02	C50 Malignant neoplasm of breast
5,004(3.28)	49(5.37)	1.73[1.10–2.73]	0.01	
3,016(3.23)	66(3.41)	1.05[0.72–1.54]	1	
3,016(3.23)	45(3.35)	1.01[0.64–1.59]	1	C51–C58 Malignant neoplasms of female genital organs
3,016(3.23)	20(3.46)	1.11[0.56–2.19]	1	
4,443(7.49)	142(11.66)	1.61[1.21–2.15]	2.05E–04	
4,443(7.49)	106(12.08)	1.68[1.22–2.31]	6.90E–04	C60–C63 Malignant neoplasms of male genital organs
4,443(7.49)	36(10.74)	1.46[0.84–2.54]	0.96	
2,632(1.72)	84(2.66)	1.56[1.11–2.2]	3.76E–03	
2,632(1.72)	53(2.39)	1.35[0.89–2.05]	0.7	C64–C68 Malignant neoplasms of urinary tract
2,632(1.72)	31(3.40)	2.16[1.23–3.78]	0.03	
1,353(0.89)	42(1.33)	1.49[0.93–2.38]	0.42	
1,353(0.89)	33(1.49)	1.65[0.99–2.76]	0.14	C69–C72 Malignant neoplasms of eye, brain and other parts of central nervous system
1,353(0.89)	8(0.88)	0.99[0.34–2.86]	1	
1,840(1.21)	66(2.09)	1.76[1.2–2.56]	9.11E–04	
1,840(1.21)	49(2.21)	1.86[1.21–2.85]	1.11E–03	C73–C75 Malignant neoplasms of thyroid and other endocrine glands
1,840(1.21)	16(1.75)	1.45[0.69–3.06]	1	
5,693(3.73)	152(4.82)	1.28[0.99–1.66]	0.09	
5,693(3.73)	104(4.66)	1.22[0.9–1.66]	0.84	C76–C79 Malignant neoplasms of ill–defined, secondary and unspecified sites
5,693(3.73)	46(5.04)	1.4[0.89–2.2]	0.64	
3,107(2.04)	91(2.88)	1.42[1.03–1.95]	0.04	
3,107(2.04)	60(2.70)	1.3[0.88–1.92]	1	C81–C96 Malignant neoplasms, stated or presumed to be primary, of lymphoid, haematopoietic and related tissue
3,107(2.04)	31(3.40)	1.74[1.01–3.01]	0.06	

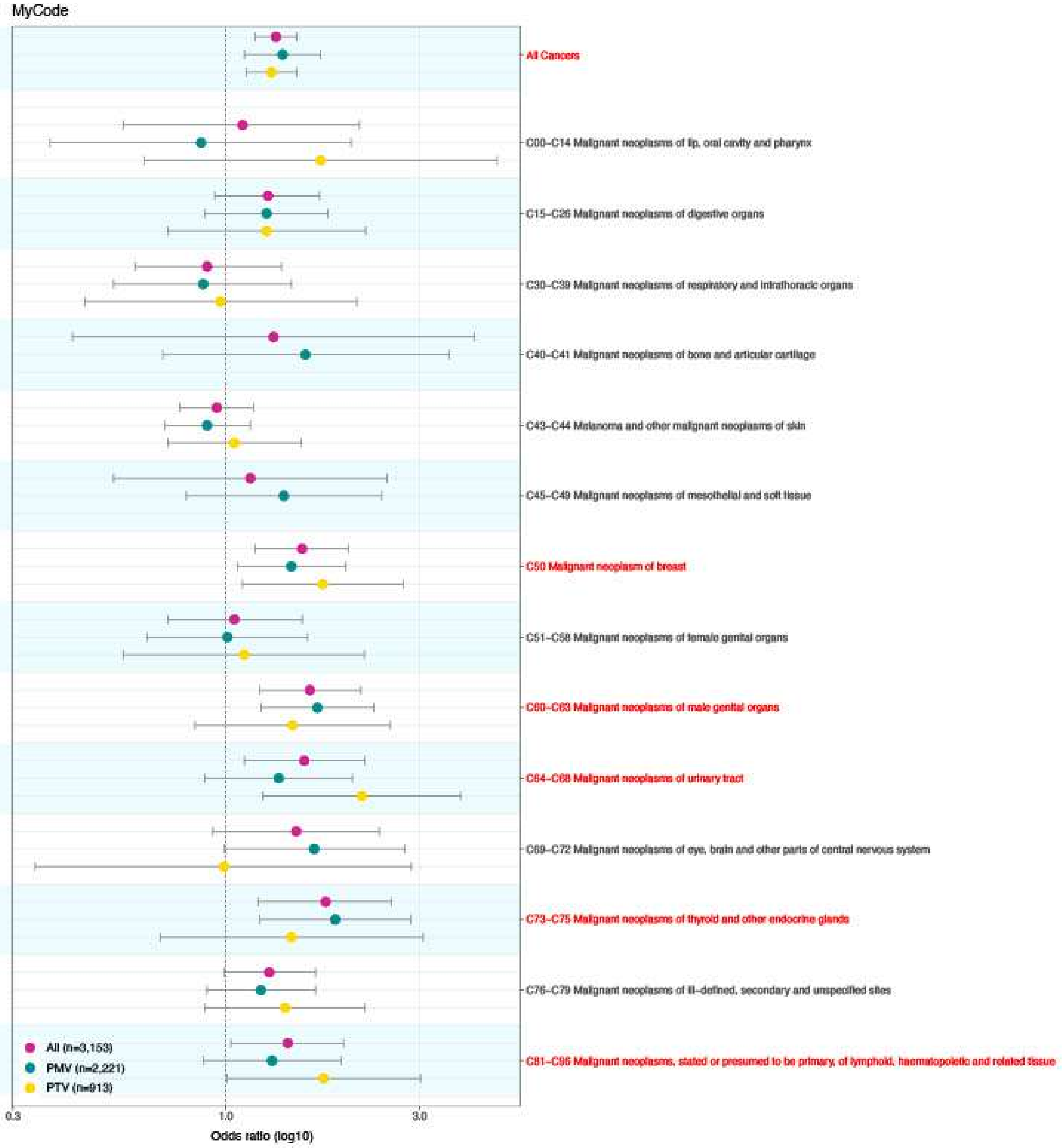

© 2025 Kim SY et al. *JAMA Network Open*.

eFigure 2. Odds Ratio for All, PTV, and PMV Groups of Case Participants for Organ System Groupings of Cancer ICD Codes in UK Biobank

Controls(%)	Heterozygotes(%)	OR [95% CI]	Adjusted SAIGE p–value
69,836(22.87)	934(28.90)	1.41[1.26–1.59]	6.05E–15
69,836(22.87)	344(26.67)	1.28[1.06–1.55]	2.19E–04
69,836(22.87)	562(30.23)	1.50[1.28–1.75]	9.56E–12
1,270(0.42)	10(0.31)	0.75[0.30–1.88]	1
1,270(0.42)	4(0.31)	NA	NA
1,270(0.42)	6(0.32)	0.78[0.24–2.57]	1
10,048(3.29)	115(3.55)	1.11[0.84–1.47]	1
10,048(3.29)	44(3.41)	1.09[0.69–1.71]	1
10,048(3.29)	66(3.57)	1.09[0.76–1.58]	1
4,169(1.37)	44(1.36)	1.03[0.66–1.61]	1
4,169(1.37)	18(1.40)	1.05[0.52–2.11]	1
4,169(1.37)	24(1.30)	0.98[0.54–1.79]	1
218(0.07)	1(0.03)	NA	NA
218(0.07)	0(0)	NA	NA
218(0.07)	1(0.05)	NA	NA
26,372(8.64)	289(8.94)	1.04[0.87–1.26]	1
26,372(8.64)	109(8.45)	1.01[0.75–1.36]	1
26,372(8.64)	171(9.26)	1.06[0.84–1.35]	1
1,169(0.38)	11(0.34)	0.82[0.33–2.08]	1
1,169(0.38)	3(0.23)	NA	NA
1,169(0.38)	8(0.43)	1.13[0.40–3.18]	1
12,439(4.07)	227(7.02)	1.84[1.49–2.27]	2.47E–12
12,439(4.07)	80(6.20)	1.55[1.09–2.19]	1.41E–02
12,439(4.07)	139(7.53)	2.05[1.57–2.69]	4.49E–10
4,047(1.33)	50(1.55)	1.20[0.79–1.83]	1
4,047(1.33)	16(1.24)	0.93[0.44–1.94]	1
4,047(1.33)	33(1.79)	1.43[0.85–2.40]	0.91
10,557(3.46)	179(5.53)	1.77[1.39–2.25]	3.64E–09
10,557(3.46)	58(4.50)	1.52[1.01–2.30]	0.14
10,557(3.46)	113(6.12)	1.87[1.38–2.53]	4.13E–07
4,351(1.43)	77(2.38)	1.75[1.24–2.46]	1.01E–04
4,351(1.43)	27(2.09)	1.58[0.89–2.80]	0.84
4,351(1.43)	49(2.65)	1.92[1.25–2.94]	2.68E–04
1,031(0.34)	14(0.43)	1.30[0.59–2.84]	1
1,031(0.34)	7(0.54)	1.66[0.55–5.02]	1
1,031(0.34)	5(0.27)	0.80[0.22–2.95]	1
773(0.25)	18(0.56)	2.23[1.11–4.49]	0.08
773(0.25)	7(0.54)	2.15[0.71–6.52]	1
773(0.25)	10(0.54)	2.19[0.86–5.56]	0.2
14,339(4.70)	192(5.94)	1.31[1.05–1.63]	4.68E–03
14,339(4.70)	66(5.12)	1.13[0.78–1.64]	1
14,339(4.70)	120(6.50)	1.43[1.09–1.90]	2.95E–03
5,030(1.65)	108(3.34)	2.11[1.58–2.82]	3.45E–09
5,030(1.65)	39(3.02)	1.95[1.21–3.15]	4.70E–03
5,030(1.65)	65(3.52)	2.19[1.51–3.17]	8.74E–07

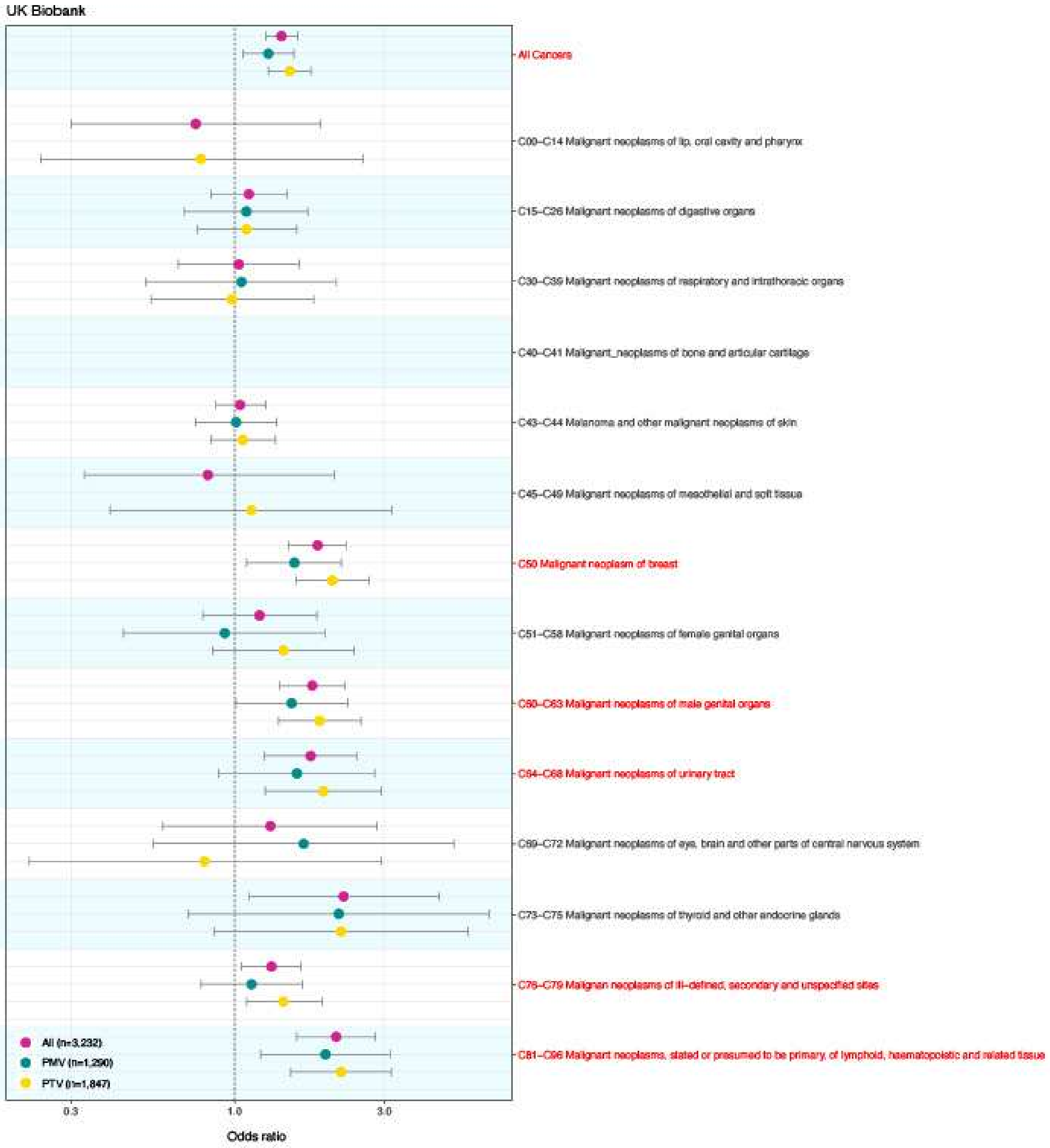

© 2025 Kim SY et al. *JAMA Network Open*.

eFigure 3. Odds Ratio for All, PTV, and PMV Groups of Case Participants for All Specific Cancers in the Organ System Groupings of Cancer ICD Codes in MyCode

Controls(%)	Heterozygotes(%)	OR [95% CI]	Adjusted SAIGE p–value
32(0.05)	1(0.08)	NA	NA
32(0.05)	0(0)	NA	NA
32(0.05)	1(0.08)	NA	NA
4222(7.12)	135(11.09)	1.62[1.27–2.07]	1.04E–04
4222(7.12)	101(11.51)	1.69[1.27–2.24]	4.03E–04
4222(7.12)	34(10.14)	NA	NA
195(0.33)	7(0.58)	1.74[0.67–4.55]	1
195(0.33)	6(0.68)	2.06[0.73–5.83]	0.43
195(0.33)	1(0.298)	NA	NA
19(0.03)	0(0)	NA	NA
19(0.03)	0(0)	NA	NA
19(0.03)	0(0)	NA	NA
1202(0.79)	39(1.24)	1.58[1.03–2.41]	0.02
1202(0.79)	27(1.22)	NA	NA
1202(0.79)	12(1.31)	1.76[0.83–3.75]	0.28
120(0.08)	4(0.13)	NA	NA
120(0.08)	1(0.05)	NA	NA
120(0.08)	3(0.33)	NA	NA
90(0.06)	3(0.10)	NA	NA
90(0.06)	3(0.14)	NA	NA
90(0.06)	0(0)	NA	NA
1477(0.96)	46(1.46)	1.50[1.01–2.23]	0.1
1477(0.96)	27(1.22)	NA	NA
1477(0.96)	19(2.08)	2.34[1.25–4.38]	3.51E–03
193(0.13)	6(0.19)	1.47[0.51–4.27]	1
193(0.13)	2(0.1)	NA	NA
193(0.13)	4(0.44)	NA	NA
1370(0.90)	57(1.81)	2.04[1.48–2.82]	1.82E–05
1370(0.90)	42(1.89)	2.15[1.47–3.14]	6.60E–05
1370(0.90)	14(1.53)	NA	NA
80(0.05)	3(0.10)	NA	NA
80(0.05)	3(0.14)	NA	NA
80(0.05)	0(0)	NA	NA
371(0.24)	6(0.19)	0.79[0.30–2.10]	1
371(0.24)	4(0.2)	NA	NA
371(0.24)	2(0.22)	NA	NA
266(0.17)	6(0.19)	1.09[0.33–3.58]	1
266(0.17)	3(0.14)	NA	NA
266(0.17)	3(0.33)	NA	NA
397(0.26)	15(0.47)	1.80[0.84–3.84]	0.5
397(0.26)	12(0.54)	NA	NA
397(0.26)	3(0.33)	NA	NA
677(0.44)	19(0.60)	1.34[0.68–2.64]	1
677(0.44)	11(0.50)	NA	NA
677(0.44)	8(0.88)	2.04[0.73–5.73]	0.87
160(0.10)	4(0.13)	NA	NA
160(0.10)	3(0.14)	NA	NA
160(0.10)	1(0.11)	NA	NA
1216(0.80)	35(1.11)	1.38[0.84–2.28]	1
1216(0.80)	24(1.08)	NA.	NA.
1216(0.80)	11(1.20)	1.55[0.64–3.75]	1
22(0.01)	0(0)	NA	NA
22(0.01)	0(0)	NA	NA
22(0.01)	0(0)	NA	NA
135(0.10)	1(0.03)	NA	NA
135(0.10)	1(0.05)	NA	NA
135(0.10)	0(0)	NA	NA
541(0.35)	13(0.41)	1.14[0.50–2.59]	1
541(0.35)	6(0.27)	NA	NA
541(0.35)	7(0.77)	2.23[0.74–6.74]	0.36
627(0.41)	27(0.86)	2.08[1.17–3.69]	7.61E–03
627(0.41)	19(0.86)	NA	NA
627(0.41)	8(0.88)	2.21[0.78–6.22]	0.53
338(0.22)	12(0.38)	1.72[0.74–4.02]	1
338(0.22)	7(0.32)	NA	NA
338(0.22)	5(0.54)	2.53[0.69–9.33]	0.57
36(0.02)	0(0)	NA	NA
36(0.02)	0(0)	NA	NA
36(0.02)	0(0)	NA	NA
35(0.02)	0(0)	NA	NA
35(0.02)	0(0)	NA	NA
35(0.02)	0(0)	NA	NA
147(0.09)	6(0.19)	1.95[0.58–6.52]	0.3
147(0.09)	1(0.05)	NA	NA
147(0.09)	4(0.44)	NA	NA
108(0.07)	2(0.06)	NA	NA
108(0.07)	1(0.05)	NA	NA
108(0.07)	1(0.11)	NA	NA

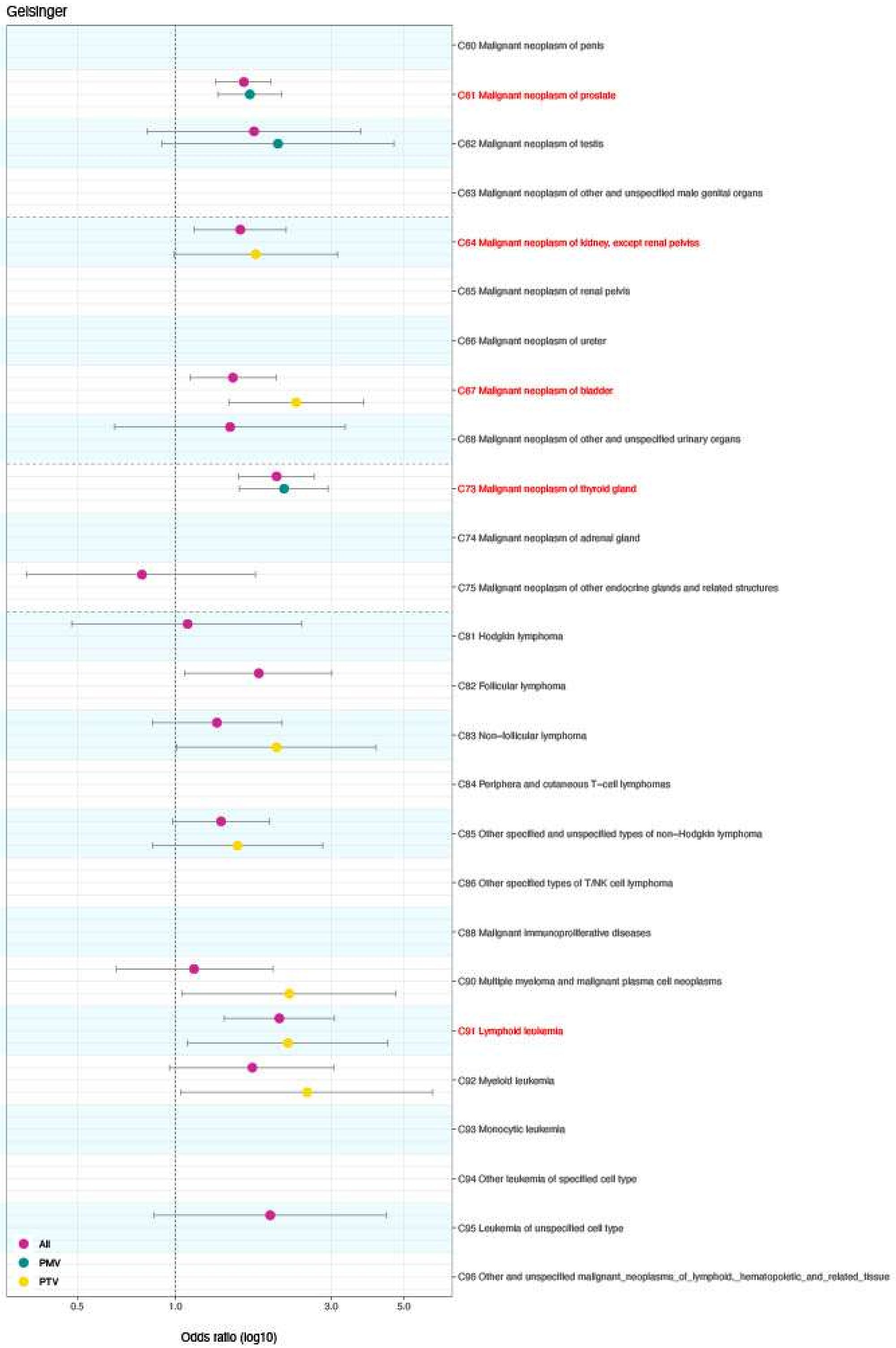

© 2025 Kim SY et al. *JAMA Network Open*.

eFigure 4. Odds Ratio for All, PTV, and PMV Groups of Case Participants for All Specific Cancers in the Organ System Groupings of Cancer ICD Codes in UK Biobank

Controls(%)	Heterozygotes(%)	OR [95% CI]	Adjusted SAIGE p–value
91(0.03)	0(0)	NA	NA
91(0.03)	0(0)	NA	NA
91(0.03)	0(0)	NA	NA
9,978(3.27)	170(5.27)	1.78[1.48–2.16]	6.68E–10
9,978(3.27)	55(4.26)	NA	NA
9,978(3.27)	107(5.79)	1.87[1.47–2.38]	1.27E–07
517(0.17)	10(0.31)	1.82[0.89–3.72]	0.12
517(0.17)	3(0.23)	NA	NA
517(0.17)	7(0.38)	2.16[0.92–5.09]	0.15
22(0.01)	0(0)	NA	NA
22(0.01)	0(0)	NA	NA
22(0.01)	0(0)	NA	NA
1,635(0.54)	31(0.96)	1.84[1.22–2.77]	2.49E–03
1,635(0.54)	11(0.85)	NA	NA
1,635(0.54)	20(1.08)	2.05[1.23–3.40]	4.28E–03
142(0.05)	2(0.06)	NA	NA
142(0.05)	2(0.16)	NA	NA
142(0.05)	0(0)	NA	NA
163(0.05)	1(0.03)	NA	NA
163(0.05)	1(0.08)	NA	NA
163(0.05)	0(0)	NA	NA
2,707(0.89)	45(1.40)	1.64[1.17–2.31]	2.66E–03
2,707(0.89)	15(1.16)	NA.	NA.
2,707(0.89)	30(1.62)	1.88[1.24–2.85]	2.32E–03
86(0.06)	3(0.09)	NA	NA
86(0.06)	1(0.08)	NA	NA
86(0.06)	2(0.11)	NA	NA
175(0.06)	3(0.09)	NA	NA
175(0.06)	1(0.08)	NA	NA
175(0.06)	2(0.11)	NA	NA
7,493(2.45)	109(3.38)	1.41[1.11–1.78]	9.77E–04
7,493(2.45)	40(3.10)	NA	NA
7,493(2.45)	66(3.57)	1.50[1.11–2.03]	4.23E–03
7,557(2.48)	94(2.92)	1.21[0.94–1.55]	0.21
7,557(2.48)	32(2.48)	NA	NA
7,557(2.48)	59(3.19)	1.32[0.96–1.81]	0.16
6,072(2.00)	84(2.61)	1.34[1.03–1.76]	0.01
6,072(2.00)	27(2.09)	NA	NA
6,072(1.99)	56(3.03)	1.56[1.13–2.17]	4.40E–03
414(0.14)	8(0.25)	1.83[0.68–4.94]	0.02
414(0.14)	0(0)	NA	NA
414(0.14)	8(0.43)	3.19[1.22–8.36]	8.40E–03
593(0.19)	10(0.31)	1.61[0.66–3.90]	1
593(0.19)	5(0.39)	2.04[0.61–6.84]	1
593(0.19)	5(0.27)	1.39[0.42–4.67]	1
1,362(0.45)	26(0.81)	1.84[1.06–3.20]	0.03
1,362(0.45)	7(0.54)	1.27[0.46–3.55]	1
1,362(0.45)	18(0.97)	2.19[1.15–4.17]	0.01
216(0.07)	6(0.19)	2.68[0.85–8.45]	0.24
216(0.07)	5(0.39)	5.76[1.70–19.50]	0.02
216(0.07)	1(0.05)	NA	NA
1,533(0.50)	29(0.90)	1.83[1.08–3.08]	0.02
1,533(0.50)	8(0.62)	1.28[0.49–3.34]	0.94
1,533(0.50)	20(1.08)	2.18[1.18–4.00]	0.02
42(0.01)	4(0.12)	NA	NA
42(0.01)	3(0.23)	NA	NA
42(0.01)	1(0.05)	NA	NA
180(0.06)	4(0.12)	NA	NA
180(0.06)	1(0.08)	NA	NA
180(0.06)	3(0.16)	NA	NA
933(0.31)	18(0.56)	1.86[0.96–3.61]	0.09
933(0.31)	7(0.54)	1.87[0.67–5.20]	1
933(0.31)	10(0.54)	1.77[0.75–4.19]	0.29
921(0.30)	21(0.65)	2.21[1.19–4.08]	0.01
921(0.30)	9(0.70)	2.44[0.99–6.05]	0.14
921(0.30)	12(0.65)	2.16[0.99–4.74]	0.08
650(0.21)	20(0.62)	2.97[1.58–5.58]	2.17E–04
650(0.21)	7(0.54)	2.66[0.95–7.41]	0.13
650(0.21)	13(0.70)	3.32[1.56–7.08]	1.22E–04
82(0.03)	2(0.06)	NA	NA
82(0.03)	2(0.16)	NA	NA
82(0.03)	0(0)	NA	NA
46(0.02)	3(0.09)	NA	NA
46(0.02)	2(0.16)	NA	NA
46(0.02)	1(0.05)	NA	NA
103(0.03)	1(0.03)	NA	NA
103(0.03)	0(0)	NA	NA
103(0.03)	1(0.05)	NA	NA
50(0.02)	5(0.16)	9.47[2.58–34.80]	1.91E–03
50(0.02)	3(0.23)	NA	NA
50(0.02)	2(0.11)	NA	NA

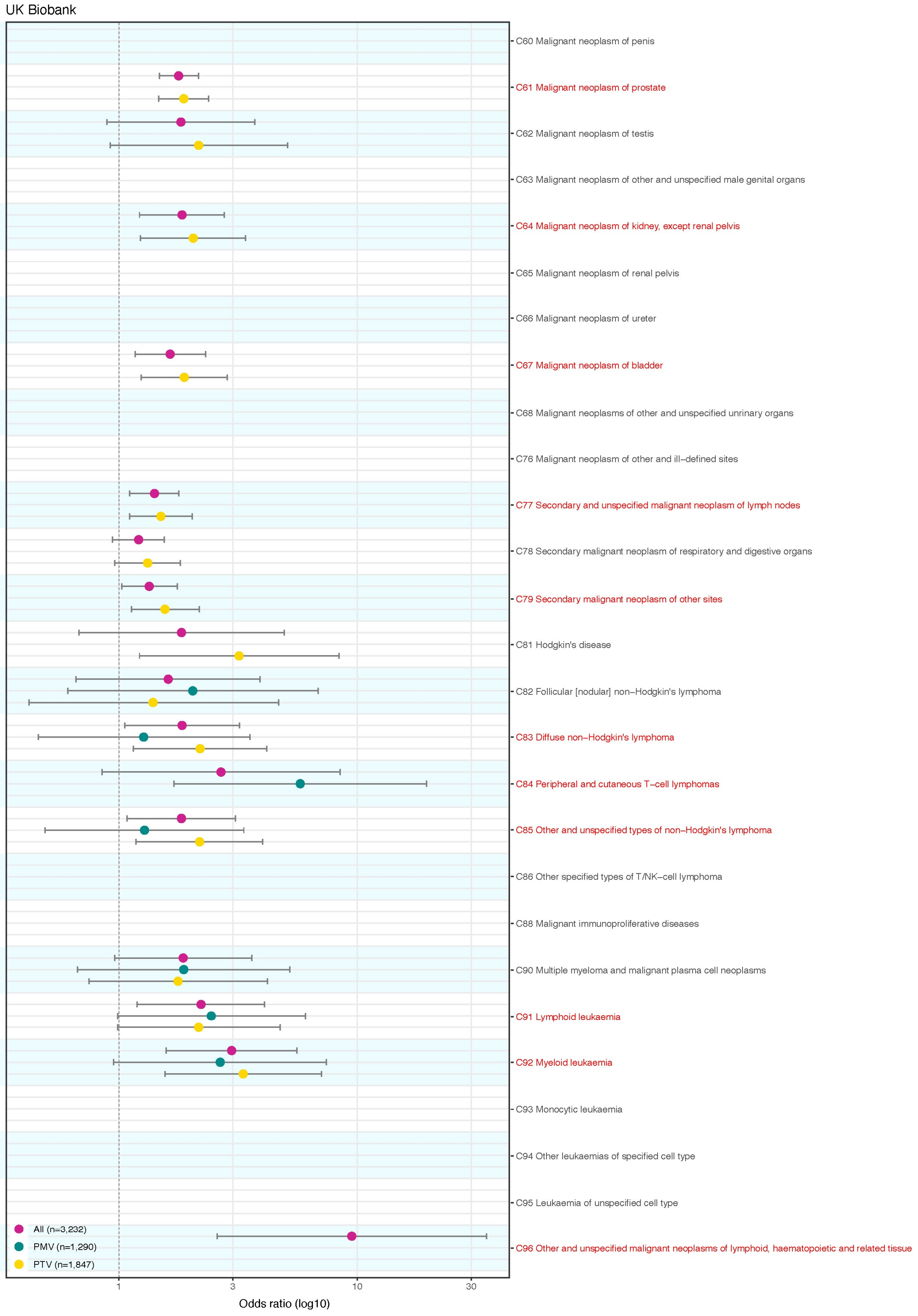

© 2025 Kim SY et al. *JAMA Network Open*.

eFigure 5. Power as a Function of Risk (Odds Ratio) in MyCode (Panel A, C, E) and UK Biobank (Panels B, D, F) for a Range of Cancer Rates

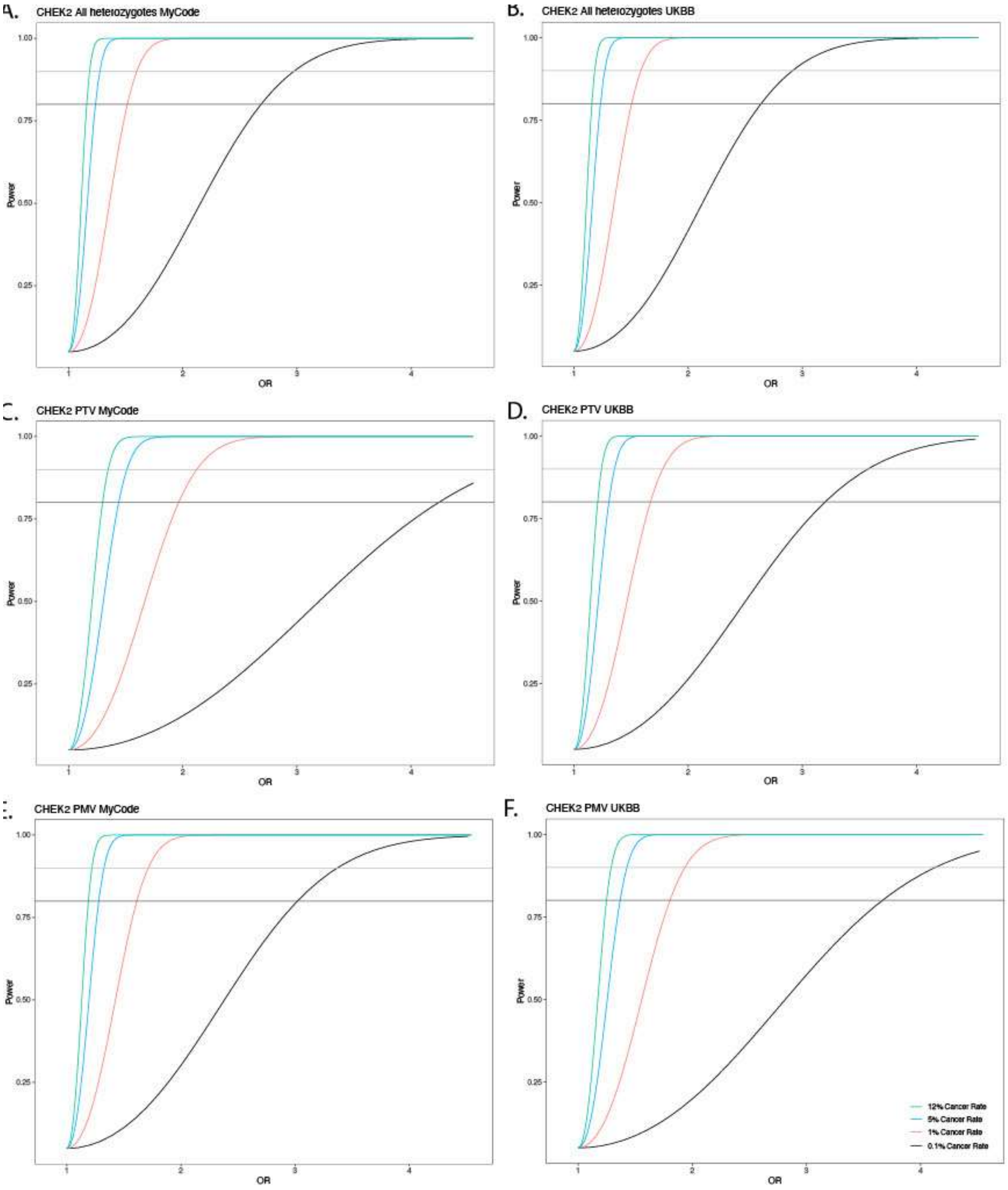

Prevalence data from cohort-specific ALL (Panels A, B) pathogenic truncating variants (PTV) (Panels C, D) and pathogenic missense variants (PMV) (Panels E, F) *CHEK2* heterozygotes

© 2025 Kim SY et al. *JAMA Network Open*.
