# Supplementary material for: Genomic Ascertainment of CHEK2-Related Cancer Predisposition
Source: JAMA Netw Open. 2025 Dec 15;8(12):e2549730. doi: 10.1001/jamanetworkopen.2025.49730 (PMC12706675; doi:10.1001/jamanetworkopen.2025.49730)
Supplement: Supplement 3. — Nonauthor Collaborators [file jamanetwopen-e2549730-s003.pdf]

Supplemental Online Content: Nonauthor Collaborators
*First name, last name, and suffix (if applicable) are required and will appear in PubMed.

*Group Name(s): Geisinger-Regeneron DiscovEHR Collaboration and Penn Medicine Biobank							
***First Name and Middle Initial(s)**	***Last Name**	***Suffix (eg, Jr, III)**	Academic Degrees	Institution	Location (city, state/province, country)	Role or Contribution, eg, chair, principal investigator	Group (if more than 1 Group listed in the byline) and/or Subgroup (eg, Steering Committee)
Aris	Baras						Regeneron Genetics Center; RGC Management & Leadership Team
Gonçalo	Abecasis						Regeneron Genetics Center; RGC Management & Leadership Team
Adolfo	Ferrando						Regeneron Genetics Center; RGC Management & Leadership Team
Giovanni	Coppola						Regeneron Genetics Center; RGC Management & Leadership Team
Andrew	Deubler						Regeneron Genetics Center; RGC Management & Leadership Team
Luca A	Lotta						Regeneron Genetics Center; RGC Management & Leadership Team
John D	Overton						Regeneron Genetics Center; RGC Management & Leadership Team

Supplemental Online Content: Nonauthor Collaborators
*First name, last name, and suffix (if applicable) are required and will appear in PubMed.

***First Name and Middle Initial(s)**	***Last Name**	***Suffix (eg, Jr, III)**	Academic Degrees	Institution	Location (city, state/province, country)	Role or Contribution, eg, chair, principal investigator	Group (if more than 1 Group listed in the byline) and/or Subgroup (eg, Steering Committee)
Jeffrey G	Reid						Regeneron Genetics Center; RGC Management & Leadership Team
Alan	Shuldiner						Regeneron Genetics Center; RGC Management & Leadership Team
Katherine	Siminovitch						Regeneron Genetics Center; RGC Management & Leadership Team
Jason	Portnoy						Regeneron Genetics Center; RGC Management & Leadership Team
Marcus B	Jones						Regeneron Genetics Center; RGC Management & Leadership Team
Lyndon	Mitnaul						Regeneron Genetics Center; RGC Management & Leadership Team
Alison	Fenney						Regeneron Genetics Center; RGC Management & Leadership Team

Supplemental Online Content: Nonauthor Collaborators
*First name, last name, and suffix (if applicable) are required and will appear in PubMed.

***First Name and Middle Initial(s)**	***Last Name**	***Suffix (eg, Jr, III)**	Academic Degrees	Institution	Location (city, state/province, country)	Role or Contribution, eg, chair, principal investigator	Group (if more than 1 Group listed in the byline) and/or Subgroup (eg, Steering Committee)
Jonathan	Marchini						Regeneron Genetics Center; RGC Management & Leadership Team
Manuel	Allen Revez Ferreira						Regeneron Genetics Center; RGC Management & Leadership Team
Maya	Ghoussaini						Regeneron Genetics Center; RGC Management & Leadership Team
Mona	Nafde						Regeneron Genetics Center; RGC Management & Leadership Team
William	Salerno						Regeneron Genetics Center; RGC Management & Leadership Team
Cristen	Willer						Regeneron Genetics Center; RGC Management & Leadership Team
Lourdes	Crane						Regeneron Genetics Center; RGC Management & Leadership Team
Christina	Beechert						Regeneron Genetics Center; Sequencing & Lab Operations

Supplemental Online Content: Nonauthor Collaborators
*First name, last name, and suffix (if applicable) are required and will appear in PubMed.

***First Name and Middle Initial(s)**	***Last Name**	***Suffix (eg, Jr, III)**	Academic Degrees	Institution	Location (city, state/province, country)	Role or Contribution, eg, chair, principal investigator	Group (if more than 1 Group listed in the byline) and/or Subgroup (eg, Steering Committee)
Erin	Fuller						Regeneron Genetics Center; Sequencing & Lab Operations
Laura M	Cremona						Regeneron Genetics Center; Sequencing & Lab Operations
Eugene	Kalyuskin						Regeneron Genetics Center; Sequencing & Lab Operations
Hang	Du						Regeneron Genetics Center; Sequencing & Lab Operations
Caitlin	Forsythe						Regeneron Genetics Center; Sequencing & Lab Operations
Zhenhua	Gu						Regeneron Genetics Center; Sequencing & Lab Operations
Kristy	Guevara						Regeneron Genetics Center; Sequencing & Lab Operations
Michael	Lattari						Regeneron Genetics Center; Sequencing & Lab Operations
Alexander	Lopez						Regeneron Genetics Center; Sequencing & Lab Operations
Kia	Manoochehri						Regeneron Genetics Center; Sequencing & Lab Operations

Supplemental Online Content: Nonauthor Collaborators
*First name, last name, and suffix (if applicable) are required and will appear in PubMed.

***First Name and Middle Initial(s)**	***Last Name**	***Suffix (eg, Jr, III)**	Academic Degrees	Institution	Location (city, state/province, country)	Role or Contribution, eg, chair, principal investigator	Group (if more than 1 Group listed in the byline) and/or Subgroup (eg, Steering Committee)
Prathyusha	Challa						Regeneron Genetics Center; Sequencing & Lab Operations
Manasi	Pradhan						Regeneron Genetics Center; Sequencing & Lab Operations
Raymond	Reynoso						Regeneron Genetics Center; Sequencing & Lab Operations
Ricardo	Schiavo						Regeneron Genetics Center; Sequencing & Lab Operations
Maria	Sotiropoulos Padilla						Regeneron Genetics Center; Sequencing & Lab Operations
Chenggu	Wang						Regeneron Genetics Center; Sequencing & Lab Operations
Sarah E	Wold						Regeneron Genetics Center; Sequencing & Lab Operations
Manan	Goyal						Regeneron Genetics Center; Genome Informatics & Data Engineering
George	Mitra						Regeneron Genetics Center; Genome Informatics & Data Engineering

Supplemental Online Content: Nonauthor Collaborators
*First name, last name, and suffix (if applicable) are required and will appear in PubMed.

***First Name and Middle Initial(s)**	***Last Name**	***Suffix (eg, Jr, III)**	Academic Degrees	Institution	Location (city, state/province, country)	Role or Contribution, eg, chair, principal investigator	Group (if more than 1 Group listed in the byline) and/or Subgroup (eg, Steering Committee)
Sanjay	Sreeram						Regeneron Genetics Center; Genome Informatics & Data Engineering
Rouel	Lanche						Regeneron Genetics Center; Genome Informatics & Data Engineering
Vrushali	Mahajan						Regeneron Genetics Center; Genome Informatics & Data Engineering
Sai	Lakshmi Vasireddy						Regeneron Genetics Center; Genome Informatics & Data Engineering
Gisu	Eom						Regeneron Genetics Center; Genome Informatics & Data Engineering
Krishna	Pawan Punuru						Regeneron Genetics Center; Genome Informatics & Data Engineering
Sujit	Gokhale						Regeneron Genetics Center; Genome Informatics & Data Engineering

*First name, last name, and suffix (if applicable) are required and will appear in PubMed.

***First Name and Middle Initial(s)**	***Last Name**	***Suffix (eg, Jr, III)**	Academic Degrees	Institution	Location (city, state/province, country)	Role or Contribution, eg, chair, principal investigator	Group (if more than 1 Group listed in the byline) and/or Subgroup (eg, Steering Committee)
Benjamin	Sultan						Regeneron Genetics Center; Genome Informatics & Data Engineering
Pooja	Mule						Regeneron Genetics Center; Genome Informatics & Data Engineering
Mudasar	Sarwar						Regeneron Genetics Center; Genome Informatics & Data Engineering
Muhammad	Aqeel						Regeneron Genetics Center; Genome Informatics & Data Engineering
Xiaodong	Bai						Regeneron Genetics Center; Genome Informatics & Data Engineering
Lance	Zhang						Regeneron Genetics Center; Genome Informatics & Data Engineering
Sean	O'Keeffe						Regeneron Genetics Center; Genome Informatics & Data Engineering

Supplemental Online Content: Nonauthor Collaborators
*First name, last name, and suffix (if applicable) are required and will appear in PubMed.

***First Name and Middle Initial(s)**	***Last Name**	***Suffix (eg, Jr, III)**	Academic Degrees	Institution	Location (city, state/province, country)	Role or Contribution, eg, chair, principal investigator	Group (if more than 1 Group listed in the byline) and/or Subgroup (eg, Steering Committee)
Razvan	Panea						Regeneron Genetics Center; Genome Informatics & Data Engineering
Evan	Edelstein						Regeneron Genetics Center; Genome Informatics & Data Engineering
Ayesha	Rasool						Regeneron Genetics Center; Genome Informatics & Data Engineering
Evan K	Maxwell						Regeneron Genetics Center; Genome Informatics & Data Engineering
Boris	Boutkov						Regeneron Genetics Center; Genome Informatics & Data Engineering
Alexander	Gorovits						Regeneron Genetics Center; Genome Informatics & Data Engineering
Ju	Guan						Regeneron Genetics Center; Genome Informatics & Data Engineering

Supplemental Online Content: Nonauthor Collaborators
*First name, last name, and suffix (if applicable) are required and will appear in PubMed.

***First Name and Middle Initial(s)**	***Last Name**	***Suffix (eg, Jr, III)**	Academic Degrees	Institution	Location (city, state/province, country)	Role or Contribution, eg, chair, principal investigator	Group (if more than 1 Group listed in the byline) and/or Subgroup (eg, Steering Committee)
Lukas	Habegger						Regeneron Genetics Center; Genome Informatics & Data Engineering
Alicia	Hawes						Regeneron Genetics Center; Genome Informatics & Data Engineering
Olga	Krasheninina						Regeneron Genetics Center; Genome Informatics & Data Engineering
Samantha	Zarate						Regeneron Genetics Center; Genome Informatics & Data Engineering
Adam J	Mansfield						Regeneron Genetics Center; Genome Informatics & Data Engineering
Joshua	Backman						Regeneron Genetics Center; Analytical Genetics and Data Science
Kathy	Burch						Regeneron Genetics Center; Analytical Genetics and Data Science

Supplemental Online Content: Nonauthor Collaborators
*First name, last name, and suffix (if applicable) are required and will appear in PubMed.

***First Name and Middle Initial(s)**	***Last Name**	***Suffix (eg, Jr, III)**	Academic Degrees	Institution	Location (city, state/province, country)	Role or Contribution, eg, chair, principal investigator	Group (if more than 1 Group listed in the byline) and/or Subgroup (eg, Steering Committee)
Adrian	Campos						Regeneron Genetics Center; Analytical Genetics and Data Science
Liron	Ganel						Regeneron Genetics Center; Analytical Genetics and Data Science
Sheila	Gaynor						Regeneron Genetics Center; Analytical Genetics and Data Science
Benjamin	Geraghty						Regeneron Genetics Center; Analytical Genetics and Data Science
Arkopravo	Ghosh						Regeneron Genetics Center; Analytical Genetics and Data Science
Salvador	Romero Martinez						Regeneron Genetics Center; Analytical Genetics and Data Science
Christopher	Gillies						Regeneron Genetics Center; Analytical Genetics and Data Science

Supplemental Online Content: Nonauthor Collaborators
*First name, last name, and suffix (if applicable) are required and will appear in PubMed.

***First Name and Middle Initial(s)**	***Last Name**	***Suffix (eg, Jr, III)**	Academic Degrees	Institution	Location (city, state/province, country)	Role or Contribution, eg, chair, principal investigator	Group (if more than 1 Group listed in the byline) and/or Subgroup (eg, Steering Committee)
Lauren	Gurski						Regeneron Genetics Center; Analytical Genetics and Data Science
Eric	Jorgenson						Regeneron Genetics Center; Analytical Genetics and Data Science
Tyler	Joseph						Regeneron Genetics Center; Analytical Genetics and Data Science
Michael	Kessler						Regeneron Genetics Center; Analytical Genetics and Data Science
Jack	Kosmicki						Regeneron Genetics Center; Analytical Genetics and Data Science
Adam	Locke						Regeneron Genetics Center; Analytical Genetics and Data Science
Priyanka	Nakka						Regeneron Genetics Center; Analytical Genetics and Data Science

Supplemental Online Content: Nonauthor Collaborators
*First name, last name, and suffix (if applicable) are required and will appear in PubMed.

***First Name and Middle Initial(s)**	***Last Name**	***Suffix (eg, Jr, III)**	Academic Degrees	Institution	Location (city, state/province, country)	Role or Contribution, eg, chair, principal investigator	Group (if more than 1 Group listed in the byline) and/or Subgroup (eg, Steering Committee)
Karl	Landheer						Regeneron Genetics Center; Analytical Genetics and Data Science
Olivier	Delaneau						Regeneron Genetics Center; Analytical Genetics and Data Science
Anthony	Marcketta						Regeneron Genetics Center; Analytical Genetics and Data Science
Joelle	Mbatchou						Regeneron Genetics Center; Analytical Genetics and Data Science
Arden	Moscati						Regeneron Genetics Center; Analytical Genetics and Data Science
Anita	Pandit						Regeneron Genetics Center; Analytical Genetics and Data Science
Jonathan	Ross						Regeneron Genetics Center; Analytical Genetics and Data Science

Supplemental Online Content: Nonauthor Collaborators
*First name, last name, and suffix (if applicable) are required and will appear in PubMed.

***First Name and Middle Initial(s)**	***Last Name**	***Suffix (eg, Jr, III)**	Academic Degrees	Institution	Location (city, state/province, country)	Role or Contribution, eg, chair, principal investigator	Group (if more than 1 Group listed in the byline) and/or Subgroup (eg, Steering Committee)
Carlo	Sidore						Regeneron Genetics Center; Analytical Genetics and Data Science
Eli	Stahl						Regeneron Genetics Center; Analytical Genetics and Data Science
Timothy	Thornton						Regeneron Genetics Center; Analytical Genetics and Data Science
Sailaja	Vedantam						Regeneron Genetics Center; Analytical Genetics and Data Science
Rujin	Wang						Regeneron Genetics Center; Analytical Genetics and Data Science
Kuan-Han	Wu						Regeneron Genetics Center; Analytical Genetics and Data Science
Bin	Ye						Regeneron Genetics Center; Analytical Genetics and Data Science

Supplemental Online Content: Nonauthor Collaborators
*First name, last name, and suffix (if applicable) are required and will appear in PubMed.

***First Name and Middle Initial(s)**	***Last Name**	***Suffix (eg, Jr, III)**	Academic Degrees	Institution	Location (city, state/province, country)	Role or Contribution, eg, chair, principal investigator	Group (if more than 1 Group listed in the byline) and/or Subgroup (eg, Steering Committee)
Blair	Zhang						Regeneron Genetics Center; Analytical Genetics and Data Science
Andrey	Ziyatdinov						Regeneron Genetics Center; Analytical Genetics and Data Science
Yuxin	Zou						Regeneron Genetics Center; Analytical Genetics and Data Science
Jingning	Zhang						Regeneron Genetics Center; Analytical Genetics and Data Science
Kyoko	Watanabe						Regeneron Genetics Center; Analytical Genetics and Data Science
Mira	Tang						Regeneron Genetics Center; Analytical Genetics and Data Science
Frank	Wendt						Regeneron Genetics Center; Analytical Genetics and Data Science

Supplemental Online Content: Nonauthor Collaborators
*First name, last name, and suffix (if applicable) are required and will appear in PubMed.

*First Name and Middle Initial(s)	*Last Name	*Suffix (eg, Jr, III)	Academic Degrees	Institution	Location (city, state/province, country)	Role or Contribution, eg, chair, principal investigator	Group (if more than 1 Group listed in the byline) and/or Subgroup (eg, Steering Committee)
Suganthi	Balasubramanian						Regeneron Genetics Center; Analytical Genetics and Data Science
Suying	Bao						Regeneron Genetics Center; Analytical Genetics and Data Science
Kathie	Sun						Regeneron Genetics Center; Analytical Genetics and Data Science
Chuanyi	Zhang						Regeneron Genetics Center; Analytical Genetics and Data Science
Sean	Yu						Regeneron Genetics Center; Analytical Genetics and Data Science
Aaron	Zhang						Regeneron Genetics Center; Analytical Genetics and Data Science
David	Corrigan						Regeneron Genetics Center; Analytical Genetics and Data Science

Supplemental Online Content: Nonauthor Collaborators
*First name, last name, and suffix (if applicable) are required and will appear in PubMed.

***First Name and Middle Initial(s)**	***Last Name**	***Suffix (eg, Jr, III)**	Academic Degrees	Institution	Location (city, state/province, country)	Role or Contribution, eg, chair, principal investigator	Group (if more than 1 Group listed in the byline) and/or Subgroup (eg, Steering Committee)
Dhruv	Shidhaye						Regeneron Genetics Center; Analytical Genetics and Data Science
Chen	Wang						Regeneron Genetics Center; Analytical Genetics and Data Science
Keyrun	Adhikari						Regeneron Genetics Center; Analytical Genetics and Data Science
Alexander	Lachmann						Regeneron Genetics Center; Analytical Genetics and Data Science
Brian	Hobbs						Regeneron Genetics Center; Therapeutic Area Genetics
Jon	Silver						Regeneron Genetics Center; Therapeutic Area Genetics
William	Palmer						Regeneron Genetics Center; Therapeutic Area Genetics
Rita	Guerreiro						Regeneron Genetics Center; Therapeutic Area Genetics
Amit	Joshi						Regeneron Genetics Center; Therapeutic Area Genetics

Supplemental Online Content: Nonauthor Collaborators
*First name, last name, and suffix (if applicable) are required and will appear in PubMed.

***First Name and Middle Initial(s)**	***Last Name**	***Suffix (eg, Jr, III)**	Academic Degrees	Institution	Location (city, state/province, country)	Role or Contribution, eg, chair, principal investigator	Group (if more than 1 Group listed in the byline) and/or Subgroup (eg, Steering Committee)
Antoine	Baldassari						Regeneron Genetics Center; Therapeutic Area Genetics
Sarah	Graham						Regeneron Genetics Center; Therapeutic Area Genetics
Ernst	Mayerhofer						Regeneron Genetics Center; Therapeutic Area Genetics
Erola	Pairo Castineira						Regeneron Genetics Center; Therapeutic Area Genetics
Mary	Haas						Regeneron Genetics Center; Therapeutic Area Genetics
Niek	Verweij						Regeneron Genetics Center; Therapeutic Area Genetics
George	Hindy						Regeneron Genetics Center; Therapeutic Area Genetics
Jonas	Bovijn						Regeneron Genetics Center; Therapeutic Area Genetics
Tanima	De						Regeneron Genetics Center; Therapeutic Area Genetics
Luanluan	Sun						Regeneron Genetics Center; Therapeutic Area Genetics

Supplemental Online Content: Nonauthor Collaborators
*First name, last name, and suffix (if applicable) are required and will appear in PubMed.

***First Name and Middle Initial(s)**	***Last Name**	***Suffix (eg, Jr, III)**	Academic Degrees	Institution	Location (city, state/province, country)	Role or Contribution, eg, chair, principal investigator	Group (if more than 1 Group listed in the byline) and/or Subgroup (eg, Steering Committee)
Olukayode	Sosina						Regeneron Genetics Center; Therapeutic Area Genetics
Arthur	Gilly						Regeneron Genetics Center; Therapeutic Area Genetics
Peter	Dornbos						Regeneron Genetics Center; Therapeutic Area Genetics
Juan	Rodriguez-Flores						Regeneron Genetics Center; Therapeutic Area Genetics
Moeen	Riaz						Regeneron Genetics Center; Therapeutic Area Genetics
Manav	Kapoor						Regeneron Genetics Center; Therapeutic Area Genetics
Gannie	Tzoneva						Regeneron Genetics Center; Therapeutic Area Genetics
Momodou W	Jallow						Regeneron Genetics Center; Therapeutic Area Genetics
Anna	Alkelai						Regeneron Genetics Center; Therapeutic Area Genetics
Ariane	Ayer						Regeneron Genetics Center; Therapeutic Area Genetics

*First name, last name, and suffix (if applicable) are required and will appear in PubMed.

***First Name and Middle Initial(s)**	***Last Name**	***Suffix (eg, Jr, III)**	Academic Degrees	Institution	Location (city, state/province, country)	Role or Contribution, eg, chair, principal investigator	Group (if more than 1 Group listed in the byline) and/or Subgroup (eg, Steering Committee)
Veera	Rajagopal						Regeneron Genetics Center; Therapeutic Area Genetics
Sahar	Gelfman						Regeneron Genetics Center; Therapeutic Area Genetics
Vijay	Kumar						Regeneron Genetics Center; Therapeutic Area Genetics
Jacqueline	Otto						Regeneron Genetics Center; Therapeutic Area Genetics
Jose	Bras						Regeneron Genetics Center; Therapeutic Area Genetics
Silvia	Alvarez						Regeneron Genetics Center; Therapeutic Area Genetics
Jessie	Brown						Regeneron Genetics Center; Therapeutic Area Genetics
Hossein	Khiabanian						Regeneron Genetics Center; Therapeutic Area Genetics
Joana	Revez						Regeneron Genetics Center; Therapeutic Area Genetics
Kimberly	Skead						Regeneron Genetics Center; Therapeutic Area Genetics

Supplemental Online Content: Nonauthor Collaborators
*First name, last name, and suffix (if applicable) are required and will appear in PubMed.

***First Name and Middle Initial(s)**	***Last Name**	***Suffix (eg, Jr, III)**	Academic Degrees	Institution	Location (city, state/province, country)	Role or Contribution, eg, chair, principal investigator	Group (if more than 1 Group listed in the byline) and/or Subgroup (eg, Steering Committee)
Valentina	Zavala						Regeneron Genetics Center; Therapeutic Area Genetics
Jae	Soon Sul						Regeneron Genetics Center; Therapeutic Area Genetics
Lei	Chen						Regeneron Genetics Center; Therapeutic Area Genetics
Sam	Choi						Regeneron Genetics Center; Therapeutic Area Genetics
Amy	Damask						Regeneron Genetics Center; Therapeutic Area Genetics
Nan	Lin						Regeneron Genetics Center; Therapeutic Area Genetics
Charles	Paulding						Regeneron Genetics Center; Therapeutic Area Genetics
Sameer	Malhotra						Regeneron Genetics Center; Therapeutic Area Genetics
Joseph	Herman						Regeneron Genetics Center; Therapeutic Area Genetics
Michelle G	LeBlanc						Regeneron Genetics Center; Research Program Management & Strategic Initiatives

Supplemental Online Content: Nonauthor Collaborators
*First name, last name, and suffix (if applicable) are required and will appear in PubMed.

***First Name and Middle Initial(s)**	***Last Name**	***Suffix (eg, Jr, III)**	Academic Degrees	Institution	Location (city, state/province, country)	Role or Contribution, eg, chair, principal investigator	Group (if more than 1 Group listed in the byline) and/or Subgroup (eg, Steering Committee)
Nadia	Rana						Regeneron Genetics Center; Research Program Management & Strategic Initiatives
Jennifer	Rico-Varela						Regeneron Genetics Center; Research Program Management & Strategic Initiatives
Jaimee	Hernandez						Regeneron Genetics Center; Research Program Management & Strategic Initiatives
Larizbeth	Romero						Regeneron Genetics Center; Research Program Management & Strategic Initiatives
Ashley	Paynter						Regeneron Genetics Center; Research Program Management & Strategic Initiatives
Randi	Schwartz						Regeneron Genetics Center; Senior Partnerships & Business Operations
Jody	Hankins						Regeneron Genetics Center; Senior Partnerships & Business Operations

Supplemental Online Content: Nonauthor Collaborators
*First name, last name, and suffix (if applicable) are required and will appear in PubMed.

***First Name and Middle Initial(s)**	***Last Name**	***Suffix (eg, Jr, III)**	Academic Degrees	Institution	Location (city, state/province, country)	Role or Contribution, eg, chair, principal investigator	Group (if more than 1 Group listed in the byline) and/or Subgroup (eg, Steering Committee)
Anna	Han						Regeneron Genetics Center; Senior Partnerships & Business Operations
Samuel	Hart						Regeneron Genetics Center; Senior Partnerships & Business Operations
Ryan	Smith						Regeneron Genetics Center; Senior Partnerships & Business Operations
Ann	Perez-Beals						Regeneron Genetics Center; Business Operations & Administrative Coordinators
Gina	Solari						Regeneron Genetics Center; Business Operations & Administrative Coordinators
Johannie	Rivera-Picart						Regeneron Genetics Center; Business Operations & Administrative Coordinators

Supplemental Online Content: Nonauthor Collaborators
*First name, last name, and suffix (if applicable) are required and will appear in PubMed.

***First Name and Middle Initial(s)**	***Last Name**	***Suffix (eg, Jr, III)**	Academic Degrees	Institution	Location (city, state/province, country)	Role or Contribution, eg, chair, principal investigator	Group (if more than 1 Group listed in the byline) and/or Subgroup (eg, Steering Committee)
Michelle	Pagan						Regeneron Genetics Center; Business Operations & Administrative Coordinators
Sunilbe	Siceron						Regeneron Genetics Center; Business Operations & Administrative Coordinators
Daniel J.	Rader						Penn Medicine Biobank; PMBB Leadership Team
Marylyn D.	Ritchie						Penn Medicine Biobank; PMBB Leadership Team
Nawar	Naseer						Penn Medicine Biobank; Patient Recruitment and Regulatory Oversight
Giorgio	Sirugo						Penn Medicine Biobank; Patient Recruitment and Regulatory Oversight
Afiya	Poindexter						Penn Medicine Biobank; Patient Recruitment and Regulatory Oversight

Supplemental Online Content: Nonauthor Collaborators
*First name, last name, and suffix (if applicable) are required and will appear in PubMed.

***First Name and Middle Initial(s)**	***Last Name**	***Suffix (eg, Jr, III)**	Academic Degrees	Institution	Location (city, state/province, country)	Role or Contribution, eg, chair, principal investigator	Group (if more than 1 Group listed in the byline) and/or Subgroup (eg, Steering Committee)
Yi-An	Ko						Penn Medicine Biobank; Patient Recruitment and Regulatory Oversight
Kyle P.	Nerz						Penn Medicine Biobank; Patient Recruitment and Regulatory Oversight
JoEllen	Weaver						Penn Medicine Biobank; Lab Operations
Meghan	Livingstone						Penn Medicine Biobank; Lab Operations
Fred	Vadivieso						Penn Medicine Biobank; Lab Operations
Stephanie	DerOhannessian						Penn Medicine Biobank; Lab Operations
Teo	Tran						Penn Medicine Biobank; Lab Operations
Julia	Stephanowski						Penn Medicine Biobank; Lab Operations
Salma	Santos						Penn Medicine Biobank; Lab Operations
Ned	Haubein						Penn Medicine Biobank; Lab Operations
Joseph	Dunn						Penn Medicine Biobank; Lab Operations
Anurag	Verma						Penn Medicine Biobank; Clinical Informatics

Supplemental Online Content: Nonauthor Collaborators
*First name, last name, and suffix (if applicable) are required and will appear in PubMed.

***First Name and Middle Initial(s)**	***Last Name**	***Suffix (eg, Jr, III)**	Academic Degrees	Institution	Location (city, state/province, country)	Role or Contribution, eg, chair, principal investigator	Group (if more than 1 Group listed in the byline) and/or Subgroup (eg, Steering Committee)
Colleen Morse	Kripke						Penn Medicine Biobank; Clinical Informatics
Marjorie	Risman						Penn Medicine Biobank; Clinical Informatics
Renae	Judy						Penn Medicine Biobank; Clinical Informatics
Colin	Wollack						Penn Medicine Biobank; Clinical Informatics
Shefali S.	Verma						Penn Medicine Biobank; Genome Informatics
Scott	Damrauer						Penn Medicine Biobank; Genome Informatics
Yuki	Bradford						Penn Medicine Biobank; Genome Informatics
Scott	Dudek						Penn Medicine Biobank; Genome Informatics
Theodore	Drivas						Penn Medicine Biobank; Genome Informatics
