# Supplementary material for: Genomic Ascertainment of CHEK2-Related Cancer Predisposition
Source: JAMA Netw Open. 2025 Dec 15;8(12):e2549730. doi: 10.1001/jamanetworkopen.2025.49730 (PMC12706675; doi:10.1001/jamanetworkopen.2025.49730)
Supplement: Supplement 4. — Data Sharing Statement [file jamanetwopen-e2549730-s004.pdf]

Data Sharing Statement

Kim. Genomic Ascertainment of CHEK2-Related Cancer Predisposition. *JAMA Netw Open*. Published December 15, 2025. doi:10.1001/jamanetworkopen.2025.49730

Data
Data available: Yes
Data types: Deidentified participant data
How to access data: The data supporting the findings of this article are reported in the main text, figures and tables. Data to reproduce the results are available to qualified academic non-commercial researchers under a data access agreement with Geisinger.
When available: With publication

Supporting Documents
Document types: None

Additional Information
Who can access the data: The data supporting the findings of this article are reported in the main text, figures and tables. Data to reproduce the results are available to qualified academic non-commercial researchers under a data access agreement with Geisinger.
Types of analyses: The data supporting the findings of this article are reported in the main text, figures and tables. Data to reproduce the results are available to qualified academic non-commercial researchers under a data access agreement with Geisinger.
Mechanisms of data availability: The data supporting the findings of this article are reported in the main text, figures and tables. Data to reproduce the results are available to qualified academic non-commercial researchers under a data access agreement with Geisinger.
